# Development of Metal Complexes for Treatment of Coronaviruses

**DOI:** 10.3390/ijms23126418

**Published:** 2022-06-08

**Authors:** Hany M. Abd El-Lateef, Tarek El-Dabea, Mai M. Khalaf, Ahmed M. Abu-Dief

**Affiliations:** 1 Department of Chemistry, College of Science, King Faisal University, P.O. Box 400, Al-Ahsa 31982, Saudi Arabia; mmkali@kfu.edu.sa; 2 Chemistry Department, Faculty of Science, Sohag University, Sohag 82534, Egypt; tarekeldabea@gmail.com; 3 Chemistry Department, College of Science, Taibah University, Madinah 344, Saudi Arabia

**Keywords:** metal complexes, pandemic, coronavirus variants, SARS-CoV-2, pharmacophore, RCSB pdb (Protein Data Bank) database

## Abstract

Coronavirus disease (SARS-CoV-2) is a global epidemic. This pandemic, which has been linked to high rates of death, has forced some countries throughout the world to implement complete lockdowns in order to contain the spread of infection. Because of the advent of new coronavirus variants, it is critical to find effective treatments and vaccines to prevent the virus’s rapid spread over the world. In this regard, metal complexes have attained immense interest as antibody modifiers and antiviral therapies, and they have a lot of promise towards SARS-CoV-2 and their suggested mechanisms of action are discussed, i.e., a new series of metal complexes’ medicinal vital role in treatment of specific proteins or SARS-CoV-2 are described. The structures of the obtained metal complexes were fully elucidated by different analytical and spectroscopic techniques also. Molecular docking and pharmacophore studies presented that most of complexes studied influenced good binding affinity to the main protease SARS-CoV-2, which also was attained as from the RCSB pdb (Protein Data Bank) data PDB ID: 6 W41, to expect the action of metal complexes in contradiction of COVID-19. Experimental research is required to determine the pharmacokinetics of most of the complexes analyzed for the treatment of SARS-CoV-2-related disease. Finally, the toxicity of a metal-containing inorganic complex will thus be discussed by its capability to transfer metals which may bind with targeted site.

## 1. Introduction

The current design and development of new antiviral agents for opposing COVID-19 is engrossed mainly on reducing the effects of the virus [1,2,3,4,5,6]. Thus, it immediately needs to develop new biological pharmacophores blessed with a broad spectrum of activity and less toxicity. Metal complexes have had a major impact on the formation of metallodrugs [7,8,9,10,11]. The overview of heterocyclic compounds, which played a critical role in controlling biological activity [12,13], was the most significant achievement as in development of drug chemistry. It’s a common choice in biomedical studies for its simplicity of synthesis as well the enrollment of imidazole moieties in medicines, proteins, and enzymes. In recent years, Schiff bases have been frequently used to create metallobiosite replacements in enzymes, such as catechol oxidase [14], galactose oxidase [15], superoxide dismutase [16], phosphatase [17], cytochrome P-450 [18], urease, and phenoxazinone synthase [19], along with others. All ligands get the interesting potential to bind with transition metals to form their respective compounds of main group, lanthanide, actinide, and transition metal elements, substantially improving their properties. The azomethine (N = CH) lone pair of electrons interact in their structure is responsible for the stability of structures with metal ions [20]. Schiff bases (SBs) with an azomethine linkage (R1–CH=N–R2) have been described as potential and adjustable compounds in drug development [21]. Thousands of chemical structural variants were examined after the discovery of Schiff bases [22,23]. They were created with the goal of obtaining the highest chemotherapeutic effects, particularly for SBs with a heterocyclic ring [24,25]. With continued interest in organic synthesis, it was desirable to identify efficient and effective synthesis methods due to their wide range of pharmacological actions. Antibiotics [26], anti-inflammatory medications [27], muscle relaxants [28], ant tubercular drugs [29], and antiviral agents [30] are only a few of the drugs that contain this backbone. Unfortunately, the virus’s rapid spread resulted in a significant rate of mortality and morbidity over the globe [31]. The World Health Organization (WHO) labeled this health emergency an epidemic on 11 March 2020 and recommended different precautions to avoid illness transmission. Almost all academic, social, cultural, and political activity was delayed during the epidemic, and the world was put on lockdown to prevent the spread of infection. Throughout the year 2020, scientists worked to identify, investigate, and analyze various medications in order to find a cure for this deadly sickness [32,33,34]. For a drug to be called an anti-COVID-19 therapy, it must interact with spike proteins and limit coronavirus genetic material replication [35]. Despite the fact that several substances are now being examined in clinical studies, the best treatment option is still unknown. Metal chelates, besides being a possible therapy for COVID-19, may have other advantages. Metal ions are needed by the human body to deliver O_2_ to all body tissues through Fe in Hemoglobin. In this context, various techniques of using therapeutic complexes with metal ions as suitable COVID-19 treatment agents might be examined. A molecular docking investigation was conducted to determine the binding mode of the prepared compounds for the SARS-CoV-2 significant protease using the UAW247 crystal-structure inhibitor (PDB-ID: 6XBH). As a result, docking studies were conducted with this goal in mind. The scoring technique has been evaluated due to the data of MOE-docking, suggesting thermodynamic properties impact the data of free binding energy for the ideal atomic level management roles; results of target analytes and free binding energy for a corrected binding site of each prepared ligand enabled for an estimate the strength of complex formation among the ligand and chelates [36,37]. The unliganded protein surface of the SARS-CoV-2 basic protease (nCoV-2019, coronavirus disease 2019, COVID-19) (PDB ID: 6Y84) microstructures was utilized to suggest the likely affinity of the synthesized chemicals.

## 2. Prevalence and Epidemiology of COVID-19

COVID-19 was confidential an epidemic by WHO on 11 March 2020. In the absence of specified therapy, the pandemic COVID-19 is becoming the greatest and the most unexpected health issue of the 21st century [38]. Coronavirus was discovered in December 2019 in Wuhan, China, and has spread across the world. COVID-19 was a pandemic generated by the SARS-CoV-2 virus that impacted over 50 million people worldwide, resulting in over 1.3 million people death [39]. COVID-19 is a rapidly evolving problem that is putting a strain on existing funds, with new infections getting detected every day. Despite strict protective measures, people are also still acquiring sickness at an unmanageable rate. This pandemic has ended up causing undetected socio-economic upheaval around the world [40]. ARDS (Acute Respiratory Distress Syndrome) is the most common symptom of SARS-CoV-2 infection (ARDS) [40,41,42,43,44]. by condensing the virus into an exhaled breath and transporting it into the respiratory system, the virus can be spread from an infected individual to a non-infected person via respiratory droplets [45,46].

## 3. History of Coronaviruses

Coronaviruses are viruses that belong to the Coronaviridae family. Several branches of this family group exist in humans, and they frequently assault the upper breathing, causing moderate symptoms similar to the common cold. On the other hand, some coronaviruses can cause serious sickness, which can lead to death. The coronaviruses that cause the SARS-CoV and MERS-CoV is (Middle East Respiratory Syndrome) coronavirus are spread through animals and people and are known to cause serious sickness. SARS-CoV has a fatality rate of 10% and MERS-CoV has a mortality rate of 27%, respectively [47]. SARS-CoV was originally discovered in Asia in February 2003, and it quickly spread over the world, resulting in 774 deaths and 8098 cases [48]. SARS-CoV is disseminated by intimate relations through SARS-infected people or straight communication with an affected person’s respiratory secretions and bodily fluids. It was thought that infected people were not really infectious until symptoms developed. As a result, contact tracking was extremely effective during the SARS-CoV outbreak. The virus was easier to diagnose and contain due to the severity of the symptoms. MERS-CoV has a transmission pattern similar to SARS-CoV. It is, however, not as common as SARS-CoV. The MERS-CoV outbreak occurred around 2012, also with the majority of studies occurring in the Mideast. COVID-19 looks to be significantly more contagious than MERS-CoV and SARS-CoV, although it seems to be less fatal, as it is common for patients to have minor symptoms besides a fair prognosis. COVID-19’s significant systemic absorption presents a unique barrier for public health workers in contacting, tracing, and resolving the ongoing epidemic.

## 4. Structure of the SARS-CoV-2 Virus

The structure and genetics of the SARS Cov-2 virus have fully been discovered; nevertheless, the virus’s exact mechanism(s) of action are still being investigated [49,50,51,52,53,54]. The membrane (M), nucleocapsid (N), envelope (E), and spike (S) protein components are shared by all coronaviruses (Figure S1), [52] while the ORF region encodes nonstructural proteins, such as papain-like protease, RNA-dependent RNA polymerase, and 3-chymotrypsin-like protease [55,56]. Coronavirus entry into host cells is hypothesized to be aided by the trans-membrane spike (S) glycoprotein [57].

## 5. SARS-COV-2 Entry and Replication Mechanisms within Human Cells

The virus is transferred to people by the nose, mouth, or eyes. The spiking protein, such as the SARS-CoV-1, attaches to the Angiotensin-Converting Enzyme 2 (ACE2) receptors on type 2 pneumocytes in the lungs’ alveoli. As shown in (Figure S1) chemicals that reduce collapsing pressure and surface tension in the alveoli [58,59]. When the ACE2 receptor connects, infected cells’ proteins break the virus’s spike protein, allowing the virus to enter the infected cells. Endocytosis or cultured cell penetration through cell fusion allows the virus to enter the infected cells. Unlike most influenza viruses, which migrate to the center from inside the infected cell, the SARS-CoV-2 virus releases positive-sense RNA. Unlike most flu viruses, which move to the core from inside the infected cell, SARS-CoV-2 releases its optimistic RNA into the target cell’s cytoplasm. This RNA is transcribed into pp1ab and pp1a polyproteins. Both are necessary for the replication and transcription of RNA. Negative-sense RNA is formed when positive-sense RNA is copied with the RNA-dependent RNA polymerases. Negative-sense RNA is transcribed or duplicated to form positive-sense RNAs (that are incorporated into the genome). The membrane, spike, envelope, and nucleocapsid proteins are all formed through translating the transcribed RNAs. The ER of the infected cell transports the proteins to the Golgi apparatus, in which they are stored in capsules and rebuilt at the infected cell membrane. Exocytosis permits newly produced viruses to exit the host cell and infect other cells. As a result of this process, the host cell dies [60,61,62].

## 6. Metal Complexes as Potential Compounds to Block SARS-CoV-2 from Entering Host Cells

Our attention was drawn to a potential electromagnetic contact between both the virus surface and ACE2 surfaces in the host cells after studying surface chemistry as a significant idea for starting a connection between species to create chemical reactions [63]. There are several essential parameters that affect the viral adsorption concept: surface-active molecules of virus particles, surface hydrophilic or hydrophobic properties, water molecules in solution, fluid pH, and temperature. Scientists also observed that the overall charge of the virus, including S protein upon that virus surfaces, varies at different pH values, implying that S protein is believed to play a key role in virus adsorption onto charging surfaces. Although their goal was to illustrate the adsorption–desorption processes of the SARS-CoV-2 virus in a standard state, they believe that obtaining such data on the virus’s structure and assembling may be useful for studying the virus’s interaction with ACE2 in a physiological setting [64]. In PBS buffer solution (pH = 7.2) [65], discovered that ACE2 is negatively charged. A multitude of parameters measures the number of viruses adsorbed, including surface charge, steric conformation, size, stability, and other properties of the virus’s outer surface proteins. Prabakaran et al. presented a probable mechanism for ACE2’s activity as a SARS virus receptor in other investigations [66]. They looked at the solvent accessibility, ACE2 surface potential, and carbohydrate and hydrophobicity distribution for putative areas implicated in the SARS-CoV S-interaction glycoproteins through its receptor in their report. They discovered that the surface of ACE2’s deep channel and surrounding ridges are negatively charged, complementing the binding affinity site that is polarized. Furthermore, the hydrophobicity of patches around the charges, as well as the absence of calories at the top of ACE2, may contribute to high-affinity virus surface binding. The relatively unknown structural properties of ACE2’s and the virus’s surfaces drew our attention in the search for compounds with therapeutic potential against the SARS-CoV-2 virus. Cationic metal complexes were appealing in this regard because positively charged metal complexes may block virus receptor binding domains from binding to ACE2 in host cells, and various metal complexes have been explored for biological activities, including antiviral capabilities [67,68,69].

## 7. Investigating Metallodrugs as Candidates against COVID-19

**Figure 1:**
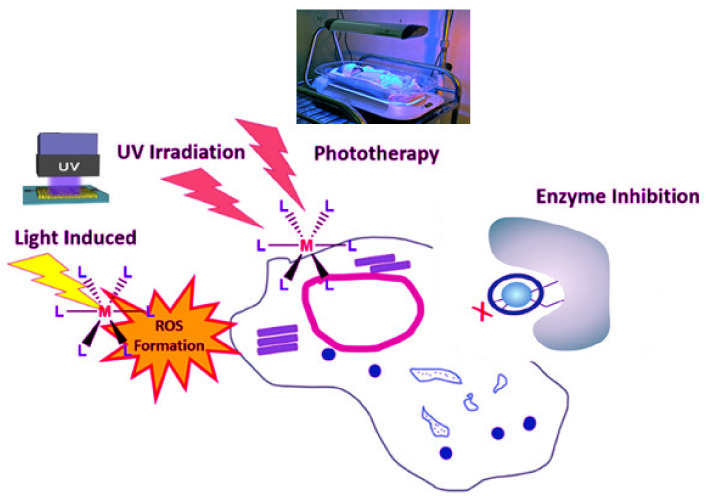
Overview of metal chelates’ mechanisms of action in living cells. Reproduced from [82].

Antiviral metallodrugs are being studied to see how they behave and what advantages they have in treating SARS CoV-2. Metallodrugs play a significant role in modern medicine. The diverse action mechanisms of these compounds, on the other hand, have only recently been discovered. We will look at some cases of the various mechanisms of action of pharmacological compounds with antibacterial and antiviral activity now. Depending on their mode of action, metal-complex medicines may be classified into various groups. Studies have sought to classify metallodrugs in this manner in a review published in January 2020 [70], offering a clear understanding of a lot of modes. During this stage, we will just discuss mechanisms that, in our opinion, could be useful in dealing with the current pandemic. Redox-active metal centers are the pathway of metallodrugs targeting virus infection, including SARS-CoV-2. Different metals may be in different positions of oxidation. The mixed valence of oxidation states may impact the kinetics of their substituents, as well as potential biological efforts in the surroundings [71,72]. We know that, in addition to extra intracellular parameters that affect the cycle time of viruses, the redox potential is a significant factor based on previous research [73]. Viruses, as we all understand, are microscopic parasites with a variety of ways of exploiting and disrupting the body’s interior for their own interest. It provides optimum settings for their functions to be carried out more effectively [74]. As a result, while infected, viruses shift the redox status towards an oxidative state [75]. It has been observed that viruses of the respiratory system can produce oxidative stress by accumulating (ROS) reactive oxygen species and around the same time dramatically deplete glutathione and the key antioxidant mechanism in the cells. These parameters, which are caused through growth in (ROS) and a reduction in the glutathione system, are extremely conducive to the virus’s reproduction pathways [76]. The activity of NOX oxidase, which consists of seven components, through the most significant, NOX2, having a critical role in virus assembly, induces over-production of ROS in infection settings. Furthermore, its absence reduces the length and severity of respiratory infections [77]. NOX4, a member of the NOX family, appears to be a much more appealing target. After infection, the NOX4 isoform is upregulated in lung epithelial cells, as a result of which reactive oxygen species are formed (ROS) [78]. The principal receptor applied by coronaviruses as access to the cell, ACE2, regulates ROS generation from NOX4. Ref. [79] ROS generation (MAPKs), in turn, activates protein kinases and enhances filial ribonucleoprotein nuclear extraction, which leads to viral replication [80]. As a result, investigations have been done that show a link between the up elevation of NOX2 and ROS of SARS-CoV-2. Damiano and her team suggest a viable treatment for such an existing pandemic by decreasing oxidative stress in cells that could reduce the number of COVID-19 patients who improve while also protecting high-risk individuals [81]. Up to this point, based on the studies, we have not found any studies of a metal-based medication that has been calculated for such ability in viruses and possesses this mode of action. The investigation and potential application of metallodrugs can change the oxidoreductase state of cells and their features, which are expected to be a logical progression in attacking COVID-19, depending upon the significance of redox potential in the virion [82]. An overview of the action mechanisms of metal chelates can be presented in (Figure 1).

## 8. Overview of Docking Studies of the Metal Complexes against COVID-19

Abbas et al. (2022) modified four additional heterocyclic of ferrocene derivatives—L1 = N-(2–hydroxy-5-biphenyl) ferrocylideneamine (Ferrocencarboxaldehyde), L2 = N-(2–hydroxy-5-nitrophenyl) ferrocylideneamine, L3 = N-(2–hydroxy-5-sulfonylphenyl) ferrocylideneamine, and L4 = N-(2–hydroxy-5-chlorophenyl) ferrocylideneamine)—which have been synthesized through reacting with an aqueous solution containing the amount of the L4, phenyl-, sulfonyl-, and nitro with 0.5 mmol ferrocenecarboxaldehyde and 0.5 mmol chloro-modified aminophenol in ethanol as just a solvent. The composition and structure of the ferrocene derivatives were identified by FT-IR, 1HNMR, electronic spectroscopy, and mass spectrometry. The composition of L4 was furthermore characterized through X-ray diffraction (single-crystal) [83]. The composition of the SARS-CoV-2 (6LU7 proteins) has been submitted to the Global Protein Database. Autodock or a model describing the MOE-docking investigation of molecules as well as controlled treatments were used to refine the (6LU7 protein) structure. Table 1 displayed the energy of binding values among ligands and proteins. It shows the binding energy relations between ligands and binding sites in the tested complexes and SARS-CoV-2 (6LU7 proteins). The interacting between hydrogen of a ligand and hydroxyl of CYS145 was determined to also be 2.40 A in L1 and 2.70 A between hydrogen of GLY143 and oxygen of the ligand. In L2, hydrogen bonding between TRP218’s keto oxygen and the ligand’s hydroxyl showed to be 2.60 A, and connection among hydrogen of ARG222 and the ligand’s O—N—O was shown to be 2.20 A. For hydrogen bonding among both the keto O of ARG279 as well as the HSO_3_ of the tested ligand has been determined as being 2.70 A in molecule L3, while the cross- linking among the keto (oxygen) of GLY278 and the HSO_3_ of the tested ligand has been determined as being 2.30 A. The molecular dynamic data for L1-L4 revealed which L1 may be more anti-COVID19 and anti-oxidant than its competitors. In comparison to the reference medications, the calculation analysis shows that L1 inhibits SARSCoV-2 more effectively. This discovery opens the way for more research into L1 and its possible use in the preventive care of SARS-CoV-2.

El-Gammal et al, (2022) synthesized (HL) the ligand of hydrazone by refluxing for 2 h in a 1:1 M ratio of the mixture of 2- Acetylpyridine (6 mmol) & (20 mL) hot ethanol of 4-(3-cyano-4,6-dimethylpyridin-2-ylamino) benzohydrazide (6 mmol) that was prepared and then acidified with glacial Ac OH drops [84,85,86]. The product was obtained and filtered, and the bright yellow filter was rinsed with Et OH before solvent extraction. This was eventually dehydrated with anhydrous CaCl_2_ in a vacuum desiccator. Cu(II) and Ni(II) and Co(II) complexes (1–3) were created by combining a hot Et OH solution of the metal salts Ni(NO_3_)_2_ 6H_2_O (1.0 mmol) and Cu(NO_3_)_2_ 3H_2_O (1.0 mmol) and Co(NO_3_)_2_. 6H_2_O (1.0 mmol), with a heated EtOH solution of HL (1.0 mmol) in a molar ratio (1 M:1 HL). Reflux for 3 h was the reaction solution. The yield was filtered off then washed with hot EtOH and dried through adding drops of petroleum ether over anhydrous CaCl_2_ and eventually dehydrated with dehydrated CaCl_2_ in desiccators [84]. The Schiff base and its Ni(II), Cu(II), and Co(II) metal complexes (1–3) as shown in (Figure S2) were characterized with different analytical and spectroscopic techniques, such as UV–Visible, FT-IR, CHN, magnetic measures, ^13^C NMR, ^1^ H NMR, magnetic, electronic spectrum, and TGA and XRD analysis. The molecular modeling using the DFT technique displays bond angles and quantum chemical factors and bond lengths and dioxygen stimulation in features of phenoxazinone included was thoroughly investigated using o-aminophenol as a standard data activated by tested complexes. The docking binding site linkages were assessed by connecting with major protease (SARS-CoV-2) that was provided from the RCSB pdb database (PDB ID: 6 W41) to predict the effect of HL in opposing COVID-19. As a result, additional preclinical and practice trials into HL as a potentially COVID-19 disease [87]. We applied docking to study the interaction of the (HL) to (6 W41). The findings suggested that HL and protein 6 W41 may be organized. The data obtained from docking relieved that a favorable interaction between HL and 6 W41 proteins target (Figure S2) with the energy calculated in (Table 2). The HB plot’s curve shows that there are intra-molecular hydrogen contacts between (HL) and the sensor, as well as reduced binding energies (kCal/mole) between both the COVID-19 (6 W41) binding site and HL. The computed results are acceptable. When AutoDock’s Ki value was matched to an experiment Ki value, Gibbs was negative. The binding site between the HL and (6W41) protein surface of COVID-19 used H-bonding via electrostatic and van der Waals interactions, with the HL exhibiting a binding affinity of –5.88 and the H-bond exhibiting binding energy of –5.88. Associations with protein residues and tested complex scoring were revealed to be possible (HL) and inhibitors of the 6W41-main proteins with just a restricted access protein surface COVID-19. As a result, we can deduce that HL has the minimum binding affinity and highest binding ability (–5.17 and –5.88 kCal/mol, respectively) [87].

Cirri et al. (2020) revealed that metal-based medicines have a wide range of chemical structures and relativities that are strictly related to the nature of the metal core and the surrounding ligands. Because of their wide chemical diversity, metal compounds are clearly a rich source of novel medicinally beneficial substances; as a result, we strongly propose that metal-based medications be included as much as possible in new drug development screening programs. This may be especially important in the hunt for innovative drugs to treat COVID-19 disease, a severe and quickly spreading disease for which medicinal treatments are currently unavailable and desperately needed. There are two basic approaches to developing metal-based medicines as anti-COVID-19 agents: medication repurposing and new drug development. Regarding the first approach, some extremely encouraging in vitro results for auranofin, a gold compound in clinical use for the treatment of rheumatoid arthritis, have been described. Similarly, several bismuth compounds appear to be highly promising. The second technique is, undoubtedly, more complex and time-intensive, but it may provide more options; it may be aided by pathway-driven or target-driven discovery considerations. The advantages and disadvantages of the two drug-discovery methodologies are examined and debated [88]. In this context, the effectiveness of early micronutrient supplementation, with an emphasis on zinc, selenium, and vitamin D, in reducing COVID-19 escalation has also been revised [89].

**Figure 2:**
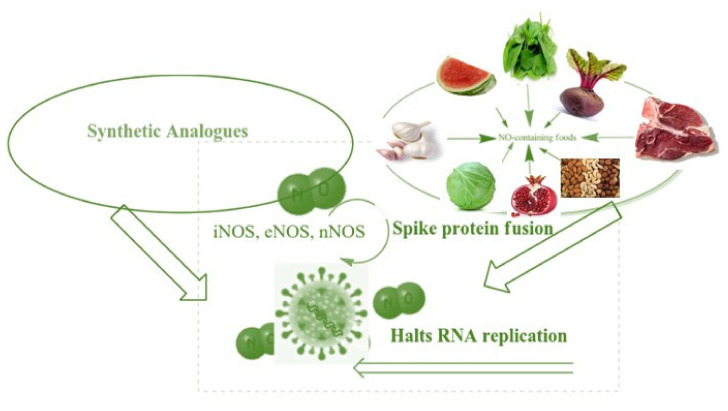
Anti-coronavirus activity of NO as inorganic ligand. Reproduced from ref. [93].

Mir et al. (2021) investigated the search for possible preventative and therapeutic antiviral techniques that are of special and urgent relevance in light of the growing COVID-19 pandemic caused by the SARS-CoV-2 virus. Zinc is known to impact antimicrobial and antioxidant immunity as well as regulate the late inflammatory response [90]. Meanwhile, the intensity of COVID-19 is linked to the problems listed above, for which Schiff base complexes have been found to be excellent candidates. However, as recently shown (Figure 2) the participation of co-ligands, such as CO, NO, and H_2_S, may enhance anti-coronavirus activity [91,92]. To consider a substance as an anti-covid medication, stimulation receptor interaction and slowing replication of coronavirus genetic code are required. Consequently, complexes depend on the nature of metals may play a significant role in the development of metallodrugs to treat this infection [93].

Ahmed et al. (2022) combined 2,2-(ethylenedioxy)bis(ethylamine) with imidazole-2-carboxaldehyde in a 1:2 ratio for the preparation of Schiff base ligand and its metal chelates with Fe(III), Mn(II), Cr(III), Ni(II), Co(II), Cd(II), and Cu(II). The metals were prepared in a 1:1 ratio. The ligand and its complexes were identified using a variety of approaches. To help further comprehend the complicated structure, classification performances, for example, FT-IR, UV, and 1H NMR spectral testing, CHN, magnetic characteristics, conductivity, TGA, BET surface area, and theoretically by DFT were applied. Docking studies in the drug development process, docking is a suitable system for predicting the good stability of a receptor–ligand interaction in order to better identify interaction features. This strategy is commonly used in the early stages of drug design and development as a virtual searching technique [94,95,96]. The researchers wanted to observe how the complexes interacted with the crystal structure of the SARS-CoV-2 basic protease with an unliganded active site (2019-nCoV, coronavirus illness 2019, COVID-19) (PDB ID: 6Y84) proteins. The compounds reacted strongly with the protein’s acceptors. When we used 6Y84 docking to examine the activation energies of binding (G) Fe(III), Mn(II), Cr(III), Ni(II), Co(II), Cu(II), and Cd(II), the best results revealed that the free energies of binding (G) were, −7.6, −6.0, −10.1, −5.9, −8.5, −7.7, −4.6, and −6.1 kcal/mol, respectively. With 3.03, 3.07, 2.99, 3.06, 2.98, 302, 2.95, and 3.07 Å distances, all tested compounds connect over H-donor, excepting ligand, which binds through H-acceptor [97,98]. Our molecules have high binding energies, indicating that may affect the metabolic changes of this protein. (Mn(L) H_2_O Cl) Cl. 3H_2_O had the strongest binding while having the lowest energy (−10.10 kcal/mol) [99,100].

Ghasemi et al. (2021) synthesized two novel mixed-ligand complexes by using a NN’O type antisymmetric tridentate ligand as the major ligand with pyridine (1) or 2,2 -dimethyl-4,4—bithiazole (BTZ) (2) as co-ligands. All crystal structures were identified with various analytical and spectroscopic techniques. Docking of macromolecules was employed to investigate how well the tested compounds interacted with 6LU7 as the receptor protein. Comparing the relative energy levels (ΔG binding) displayed that complex (2) was more efficient than (1) and favipiravir as a reference drug. Such binding energies were high quality (2) > (1) > (SB) > (BTZ). This shows the surface of the protein receptor interacted between both the tested complexes and favipiravir with 6LU7. Three hydrogen bonding relating a few of the NH_2_ group’s H atoms, and also the H15 and H16 to ASN142, and GLU166, were discovered at 1.91, 2.12, and 2.06 in complex (1). C–H bonding was also discovered at 3.24 with C pyridine and ASN142. At 5.24, alkyl hydrophobic connecting in between phenyl ring from of the aldehyde molecule and MET165 was discovered, as is another alkyl hydrophobic connection to HIS163. The interaction between Br and ASP 187 was estimated to be 3.53. At 2.69, 2.51, 2.22, 2.20, 2.13, and 2.10, six hydrogen bondings among NH_2_, H18, and H19 to ASN142, GLU166, and BTZ were identified in complex (2). Around 4.88 a d 5.73 Å, two π -sulfur bondings to HIS163 and HIS172 were discovered, as well as two π -alkyl polar bondings to HIS41 at 4.51 and 5.38 Å. Five hydrogen bondings among oxygen and hydrogen ligand atoms to SYS156, LYS100, ASP33 (two), and N of ligand have been discovered at 3.73, 1.77, 2.00, 1.69, and 1.77 Å. In the (SB) ligand at 2.99 Å, one π -lone pair-bonding in between the ligand ring and TYR101 was identified. At 4.24 and 4.75 Å, one alkyl Hyd and π -alkyl Hyd to LYS102 were discovered. The C–H bond between the N atom and VAL18 was determined to be 3.78 Å in the (BTZ) ligand. At 2.37 Å, one π -donor-H bond was discovered between such a ring of ligand and GLY71. A single π -lone pair connection to MET17 was discovered. At 3.49 and 5.40 Å, two alkyl Hyd. bonding here between C atom and ALA70 and VAL18 were discovered. At 4.71 and 4.77 Å, two π-alkyl Hyd. links to ALA70 and TRP31 were discovered. All of the analyzed materials [101,102] had a visual analysis of molecular docking, which involved drugs–receptor H. connection contacts, 2D-ligand–receptor hydrogen bond connection, and receptor side surfaces hydrogen bond connection. These tested complexes have greater negative binding energies than other tested Cu-complexes described in the literature [103], which might be related to the and BTZ moieties and acceptor groups of the aldehyde [104].

**Figure 3:**
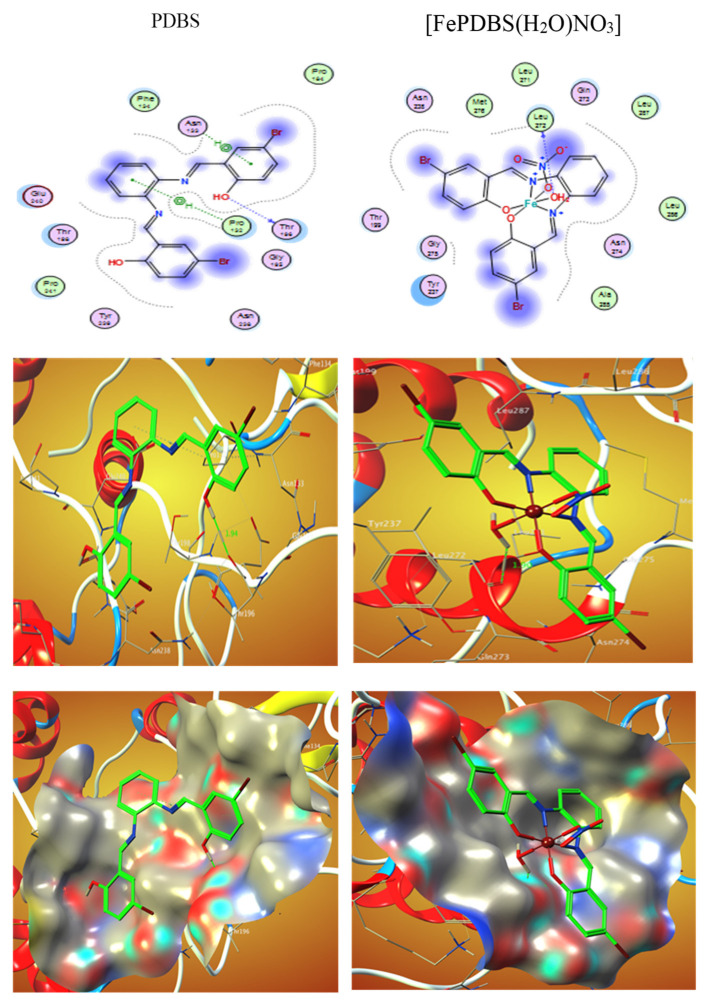
2D and 3D maps illustrating the interaction of PDBS and (FePDBS (H_2_O) NO_3_) also with the cell surface of the viral protein receptor (PDB ID: 6lu7). Hydrophobic contacts with amino acids are depicted by dashed lines.

**Figure 4:**
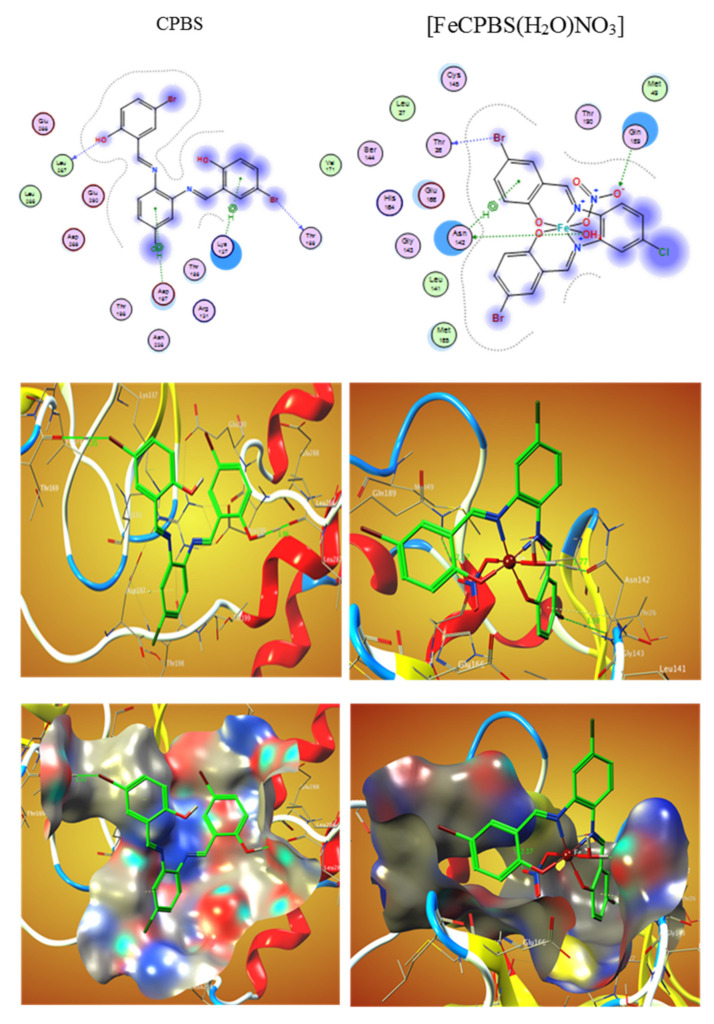
2D and 3D graphs illustrating the interaction of CPBS and (FeCPBS (H_2_O)NO_3_) with the active site of the viral protein receptor (PDB ID: 6lu7). Hydrophobic interactions with amino acid residues are depicted by dotted curves.

El-Lateef et al. (2022) prepared several salphen compounds and their Fe^3+^ studied chelates were synthesized besides fully characterized through several spectroscopic and physicochemical tools, such as CHN, IR, NMR, UV-Vis spectra, vibration spectra, magnetic moment, and TGA. Additionally, the modifications of the tested structures were studied by theoretical calculations. The relationship among all results displays that CPBS and PDBS ligands interact as tetra-dentate binding through deprotonated azomethine (two) and two-(OH) groups to form octahedral complexes with Fe^3+^ ions. The free binding energies of tested CPBS and PDBS ligands and complexes with the coronavirus receptor main protein (PDB ID: 6lu7) were reported toward being −6.9, −4.6, −18.0, and −13.3, and kcal/mol for CPBS, PDBS, (FeCPBS (H_2_O) NO_3_), and (FePDBS (H_2_O) NO_3_), respectively, as revealed in (Table 3) [105]. The further negative the binding energy, the greater the interaction. So, the connections are in the demand of PDBS < CPBS < (FePDBS (H_2_O) NO_3_) < (FeCPBS(H_2_O)NO_3_). Figure 3 and Figure 4 show 2D and 3D plots of CPBS, PDBS, (FeCPBS (H_2_O) NO_3_), and (FePDBS H_2_O)NO_3_) associations with the binding site of the coronavirus receptor protein (PDB ID: 6lu7).

Mohamed et al. (2021) used a tridentate ligand, 4-((1-(5-acetyl-2,4-dihydroxyphenyl)ethylidene)amino)-1,5-dimethyl 2-phenyl-1H-pyrazol-3(2H)-one, to form mononuclear chelates of Cr(III), Cu(II), Fe(III), Zn(II), Ni(II), and Mn(II). The tridentate coordination behavior of the ligand was demonstrated in all complexes from an azomethine N_2_ and O_2_ atoms, as indicated by IR spectrum analyses. The M to L ratio was confirmed to be 1:1, as indicated by elemental analysis and described by spectroscopic methods. For molecules and pharmaceuticals employed in prescriptions for the management of COVID-19, computer estimations of antimicrobial activities were made [105,106]. The target of the docking studies tests was to simulate the binding possibilities for H_2_L ligand and its chelates to the crystalline structure of the SARS CoV-2 major protein in association with inhibition UAW247 (6XBH). Shows the least many free binding energy of both ligand and their complexes estimated through 6XBH. The interaction models of H_2_L and their related complexes against protein were represented in 3D interaction maps. It was determined that all of the drugs tested interacted via hydrogen bonds (Table 4). Ligand, Fe(III), Cr(III), and Ni(II) complexes interact via H-donor to (Glu 166) glutamic acid ad (Met 49) methionine, whereas Cu(II), Mn(II), Zn(II), and Cd(II) complexes bind via H-acceptor to glutamine (Gln 189), glycine (Gly 143), and Glu 166, respectively, with 2.88, 3.38, 3.77, 2.86, 3.31, 3.08, 3.06, and 3.34 Å distances, respectively [106,107]. The interaction energies indicated that the Cr(III) complex has a much more constant connection and low activation energy. Then the binding energy for the novel coronavirus demonstrates substantial interactions with its targets. This could explain the formation of hydrogen bonds between Met165 and Gln166 amino acid residues. The studied complex is protected by pi-H connections with Glu189 receptors [108,109,110,111].

Alshammari et al. (2021) prepared a chain of N-substituted-2-quinolonylacetohydrazides with the goal of testing their effectiveness against SARS-CoV-2. Mass spectra, NMR, IR, and CHN all verified the structures of the observed molecules. Most of the drugs studied had a good binding affinity to the SARS-CoV-2 major protease (M^pro^), which was equivalent to Remdesivir, according to molecular docking calculations. Molecular modeling simulations have been done using Autodock software (version 4.2.6) to identify the binding mechanism and sensitivities of the prepared molecules (4a–k) of the SARS-CoV-2 main protein (Mpro). (Table 5) shows the estimated docking values and the interacting properties of compounds 4a–k with Mpro. (Table 5) shows the estimated docking tests and the binding properties of molecules 4a–k through Mpro receptor. The computed docking scores in Table 5 revealed that compounds 4a–k had good interaction binding for M^pro^, through values ranging from 7.5 to 9.7 kcal/mol. Conversely, MOE modeling of Remdesivir gave interacting energy of −8.5 kcal/mol by M^pro^ (Table 5). Assessment of the interacting abilities exposed which molecule 4d exhibited the highest interacting abilities to M^pro^ with results of −9.7 kcal/mol. The great interacting energies of molecule 4d towards M^pro^ may be qualified to generate four important hydrogen bonds with measurements of 2.07, 2.22, 2.02, and 1.83 Å with GLU166, SER144, LEU141, and HIS163 amino acids, respectively. 2D LigPlus models of contacts of molecules 4a–k interacted with vital protein sites of SARS-CoV-2 M^pro^. Overall, the molecular modeling data might test the theory that the synthesized molecules can be effective SARS-CoV-2 M^pro^ inhibitors [112].

Ali et al. (2021) prepared Cu chelates of two ligands, hydroxychloroquine, and chloroquine and described through X-ray diffraction, electronic spectra, and TGA, and these were screened using an in silico technique. More strategies and available techniques are often used in a study of the treatment and its target molecule. As an example of analyzing potential drug candidates before entering the laboratory, we use theoretical investigations on previously described biologically active complex compounds. On some COVID-19-related proteins, we performed DFT calculation studies for structure elucidation and modification, as well as molecular docking experiments. According to our findings, drug development really should be computer-aided. [113] In comparison to the parent medication, the data showed that both polar and non-polar groups within metal chelates were balanced. The metallodrug interactions here with the receptor of the viral ADP-ribose-1 monophosphatase enzyme are promoted by this structural equilibrium. As a result, the enzyme’s activity is blocked, which is the intended effect of COVID-19 infection prevention [114].

Almalki et al. (2021) used in silico analysis molecular docking to describe the production of Cu (II) and Co (II) thiazole chelates as well as the activity enhancers for contact with COVID-19 [115]. We also used CHN, electronic, and spectral studies to confirm the preparation of binuclear ligand complexes (Cu(II), Ni(II), Co(II), and Zn(II)). Molecular modeling was used to screen the generated medicinal metal complexes for biological assessments. Refat et al. (2021) showed that Ni(II) complex has higher binding effectiveness for COVID-19 protease (6LU7), according to molecular docking experiments [116]. In addition, they developed a novel COVID-19 medications method based on simulation investigations, including transition metal (Fe, Cr, and Ni) loaded fullerenes–favipiravir chelates [117].

Hecel et al. (2020) showed that Zn(II) is an immune enhancer and a virus regulator (RNA-dependent RNA polymerase inhibitor). The impact of internal Zn(II) content on both RNA and DNA viruses, mainly that affect the respiratory system, including such pi coronaviruses, and respiratory syncytial virus and influenza have been described in several investigations. One of the modes of action for chloroquine and hydroxychloroquine’s efficacy in treating COVID-19 is its ability to act as a Zn(II) ionophore, delivering more Zn(II) into the cells. Consequently, Zn(II) is one of the attractive and viable options for a suppressive effect impact on the SARS-CoV-2 replicative cycle amongst transition metals [118]. Poupaert et al. (2020) showed that various research papers back up Zn(II) as the gold standard for COVID-19 medications. The studies used quantum mechanics molecular simulations to demonstrate Zn^2+^ interactions at the molecular level. They suggest that first- and second-generation medicaments, like azithromycin (Zn^++^-antibiotic combination), could be used to treat COVID-19 [119].

**Figure 5:**
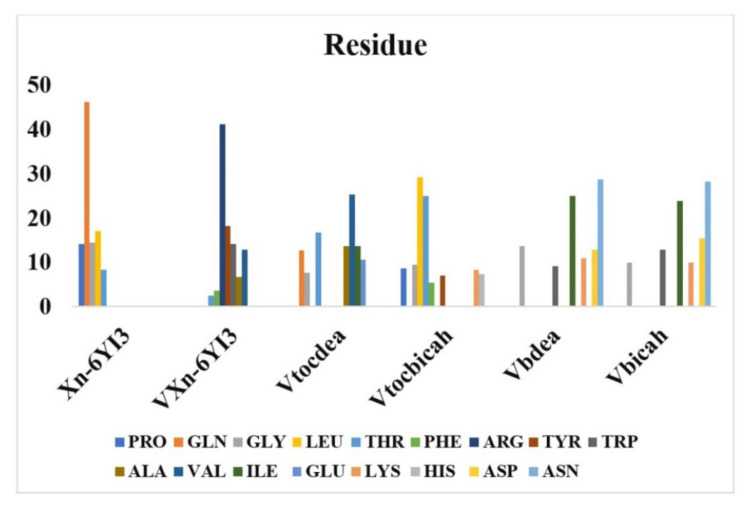
The % binding energies of 6YI3’s important amino acids on the various compounds investigated inside the paper. Reprinted/adapted with permission from Ref. [121].

M. C. Vlasiou and K. S. Pafti (2021) used one of the most widely structure-based design techniques, MOE-docking. We selected to do studies with various binding proteins to coronavirus, as described in the overview, including at least one blood transport protein so the ligand was being tested also as a therapeutic target for coronavirus. Docking tests revealed that the VXn codified receptor had the binding interactions score for BSA protein of any ligand, followed by Xn which has a binding free energy of −10.872 kcal/mol. It forms a hydrogen bridge with TYR147, ASP108, ILE455, and PRO146 (four H bonds) and interacts with BSA by van der Waals forces, which has a binding free energy of −9.323 kcal/mol. The compound Vbicah has a binding energy of −10.888 kcal/mol for the N-terminal RNA-binding domain of the SARS-CoV-2 nucleocapsid glycoprotein (6YI3), followed by VXn with −10.523kcal/mol. The maximum energy contact of an Xn with 6YI3 is connected to the amino acid glutamate, whereas the highest energy interaction of VXn through the same protein is associated with the intracellular domain. Remarkably, both Vbdeah and Vbicah exciting force with the 6YI3 amino acid asparagine. Proceeding with SARS-CoV-2 RNA-dependent polymerase (6M71), beginning through compound that has the greatest reactivity for protein, Vbicah(−10.266 kcal/mol) > Vtocdea (−9.891 kcal/mol) > Vtocdpa (−9.706 kcal/mol) > Xn (−9.603 kcal/mol) > VXn (−9.364 kcal/mol) > Vbdea (−8.647 kcal/mol). The % interaction energies of the enzyme’s active site 6YI3 on the tested compounds are shown in (Figure 5). The maximum energy interface of an Xn compound for 6YI3 is connected to the protein glutamine, whereas the greatest energy interaction of VXn with the same protein is associated with the amino acid arginine. Remarkably, both Vbdeah and Vbicah interact better with the 6YI3 amino acid asparagine. Finally, in terms of complex formation with COVID-19 basic protease (6M03), Vtocdea and VXn are among the greatest compounds. Vtocdea interacts with other organisms via hydrogen bonding. Vtocdea connects with both (SER255, and PRO322) via hydrogen bonds, while VXn connects with the proteins (GLN110, and THR111) via hydrogen bonds. The electrostatic interactions had a limited impact on such connections. Surface charges are only visible between Vtocdea and 6M03 (−0.029 Kcal/mol), Vtocdpa and BSA (−0.162 Kcal/mol), Vtocdpa and 6M71 (−0.294 Kcal/mol), Vtocdea and BSA (0.042 Kcal/mol), and Vtocdpa and 6M03 (−0.018 Kcal/mol). The electrostatic attraction of Vtocdea and Vtocdpa is consistent with previous research [113,120], demonstrating that these compounds have anti-cancer potential by disrupting the charge transfer series in mitochondria. Considering the theoretical research, we believe that compounds like Vtocdea and VXn are promising candidates for in vitro and in vivo testing of the COVID-19 virus [121].

**Figure 6:**
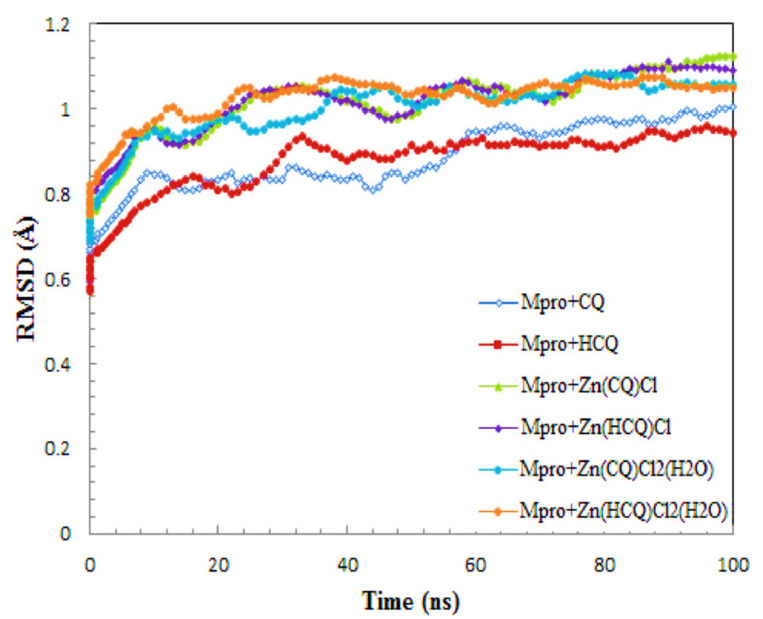
The RMSD charts for Mpro-complexes derived from molecular dynamics simulations. Reprinted/adapted with permission from Ref. [122].

R. K. Hussein and H. M. Elkhair (2021) presented various efforts made to identify therapeutic prescriptions for treating this virus. As a result of the absence of a significant publication for such a suitable therapy to treat COVID-19, the use of zinc chelates as a supplement to chloroquine/hydroxychloroquine in the treatment of COVID-19, the investigations show highlighted molecular docking and molecular dynamics methodology as effective tools for developing effective treatments to control the COVID-19 pandemic. Some zinc-CQ/HCQ complexes have still been identified to target COVID-19’s Mpro basic protease. According to molecular docking simulations, Zn(QC)Cl_2_(H_2_O) and Zn(HQC)Cl_2_(H_2_O) had the lowest binding affinity (−7.70 Kcal/mol and −7.54), respectively. The hydrogen bonds detected have been in the greatest range of H Bond group distances. The analysis of binding site contacts indicated that Zn(QC)Cl_2_(H_2_O) forms 3 hydrogen bonds with the basic protease receptor, but Zn(HQC)Cl_2_(H_2_O) forms 8 hydrogen bonds with the basic protease receptor. The observed study of RMSD and RMSF pathways in MOE dynamics simulations revealed the remarkable binding mode and physical properties of Mpro-Zn(CQ/HCQ) Cl_2_H_2_O chelates. It is possible to deduce that Zn (CQ/HCQ) Cl_2_H_2_O has a high potential as a COVID-19 Mpro inhibitor, as represented in Figure 6 [122].

## 9. In Vitro and In Vivo Potency of Metallo-Compounds against SARS-CoV-2

Yuan et al. (2020) employed metal chelates as antibiotic agents in the past. Unfortunately, their antiviral properties have not been well investigated. Bismuth medicines and similar compounds have been exposed to have strong antivirus effectiveness against SARS-CoV [123] in previous experiments. On the basis of these findings, we chose six metal compounds: two bismuth(iii) (Bi(TPP) (TPP, tetraphenylporphyrinate) and two bismuth(iii) citrate-based drugs (colloidal bismuth subcitrate (CBS) and RBC) and Bi(TPyP) (TPyP, tetra(4-pyridyl) porphy. As represented in (Figure S2). In monkey kidney Vero E6 cells, the (CC50) 50% cytotoxic activity concentrations of the tested complexes were calculated to be 2243 ± 43 μM for RBC, 3254 ± 21 μM for CBS, >400 M for both Bi(TPyP) and Bi(TPP), 13.5 ± 1.8 μM for Au(PEt3) Cl, and 14.2 ± 1.3 μM for auranofin. Because of their auspiciously lower cytotoxicity compared to Au(i)-based therapies, the four bismuth(ii) molecules were picked for more examination on potential CC50 rates in human colorectal Caco-2 cells, relieving similar CC50 data rates greater from 400 to 3740 M. As represented in Figure S3. The ½ efficient dosages (EC50) of the bismuth(iii) complexes were evaluated at little µm values and then were observed to be 2.3 ± 0.5 µM in RBC and 4.6 ± 0.4 µM and CBS, 7.5 ± 0.9 µM for Bi(TPyP), and 3.9 ± 1.2 µM for Bi(TPP). Interestingly, adding all four bismuth(ii) complexes to Caco-2 cells and Vero E6 at 1 h after infection (h.p.i.) reduced viral genome levels in a daily dosage mode (Figure S3). RBC and CBS had more strong anti-coronavirus activity than Bi(TPyP) and Bi(TPP) at non-toxic doses, as demonstrated by the optimum ~2-log versus 1-log dosage decreased in Vero E6 cell suspension Figure S3a–d, ~3-log towards ~2-log decrease in Caco-2 cell suspension Figure S3e–h, and ~4-log towards ~3-log decrease in Vero E6 and Caco-2 cell lines medium Figure S3i–l,m–p. Interestingly, Bi(iii) drugs/complexes significantly reduced SARS-CoV-2, also demonstrated through significantly lower virus nucleocapsid production in drug-treated control cells in comparison to DMSO-treated cells Figure S4a–f. Toward found out that stages of the SARS-CoV-2 transcription and replication were disrupted through drug complexes, which used a period test, which involved handling virus-infected cells for each complex at various intervals, then assessing viral titer after 9 h post-infection, once the first round of virus replication was observable in the culturing medium. Figure S4g,h. Bi(TPyP) was found to considerably inhibit virus reproduction during co-incubation of cells, but no action was seen after Bi(TPyP) was sustained once virus assembly, that Bi(TPyP) may interact with SARS-CoV-2 attachment to the surface of the cell. When RBC was added during the pre-incubation step, it had no effect on virus replication, but when it was added during the co-incubation and post-entry periods, it reduced viral loads by ~2 log, implying that RBC is a multi-target therapy that acts during virus entrance or initial occurrences when virus replication. The inhibition of virus entry by RBC and Bi(TPyP) was verified through a system that gave a virus infection test, which revealed that RBC and Bi(TPyP) reduced the percentage of virus entrance by ~45% and 53%, respectively (Figure S4i). Both CBS and Bi(TPP) work correctly only at the post-entry stage. Briefly, SARS-CoV-2 shows a sensitive effect on therapy with bismuth(ii)-based medicines [124].

Grazia Martina et al. (2021) estimated the antiviral potency of the tested molecules towards coronavirus, a DYRA, association with cells infection in the appearance of repeated therapy dilutions, has been employed as earlier defined, towards slight variations. The bithiazole chemotype is a promising substrate for the design of BSAAs by targeting the host kinase machinery (PI4KIII) or certain targets, as seen here with SARS-CoV-2. As detailed, the bithiazole chemotype can be modified to produce multitarget equivalents that act on many targets involved in various diseases (for example, cystic fibrosis and associated viral infections). The findings in this research may serve to assist the development of tailored bithiazoles with increased broad-spectrum antiviral efficacy by effectively inhibiting various targets involved in virus propagation. More investigations upon the class of BSAAs are being carried out to analyze their mechanism of action and antiviral potential [125].

Vicenti et al. (2020) found that anti-Zika (ZIKV) and anti-DENV virus medication development can be accelerated by using practical cell-based assays. Using a pan-flaviviral monoclonal antibody that recognizes a conserved envelope domain, we designed an immunodetection assay (IA). The final procedure incorporates a direct virus yield reduction assay (YRA) in the human Huh7 cell line, followed by supernatant transfer to a separate Huh7 culture to assess late antiviral effects. The IC_50_ values for sofosbuvir and ribavirin determined in the secondary YRA were repeatable and identical to those obtained in the direct YRA and plaque reduction assay (PRA). Celgosivir was efficacious against DENV only in the second YRA (IC_50_ 11.0 1.0 µM) and PRA (IC_50_ 10.1 1.1 µM), consistent with the suggested mechanism of late action. The assay format solves significant limitations of the gold standard PRA by allowing continuous investigation of potential antiviral drugs against multiple viruses and provides initial data on early versus late antiviral activity. 25,000 Calu-3, pre-seeded in the 96-well plates, were incubated by repeated various concentrations of both tested molecules and cultivated at 37 °C through 5% CO_2_. The pathogen sample was introduced at a dose of 250 PFU/well, then after 1 h of adsorbed, the media was withdrawn, and new various concentrations of each tested compound were applied to the cultures. An immune detection assay (IA) was used to measure antimicrobial activities on cell monolayers after a 48-h incubation at 37 °C with 5% CO_2_ [126]. The IA consisted of cell fixation and inhibiting the synthesis, proceeded by 1-h culturing through a specific SARS Ribonucleoprotein. Protein antibody (Milano, cat, Italy, Novus, AP201054), diluted 1:1000 in blocking buffer (PBS) enclosing. After cleaning, structures were treated over 1 h with a monoclonal HRP-coupled anti-mouse IgG tested molecule diluted 1:5000 with delaying buffer (NB7570, Italy, Novus Bio, Milano). After cell wash, the 3, 30, 5, 50-Tetramethylbenzidine substrate (Sigma Aldrich, Darmstadt, Germany) was applied to each well, followed by one liter of sulfuric acid 0.5 M to terminate the reaction. The Absorption Coefficient Module of the GloMax^®^ exploring Multimode fiber 96-well plate Reader was used to evaluate absorbance around 450 nm optical density (OD450) (Promega). Molecules that have been inactive in DYRA were, subsequently, tested in SYRA to see if they had any late antiviral effects. SYRA was carried out, employing a methodology for SARS-CoV-2 that had previously been described [126].

Cirri et al. (2021) carried a homogenates microbes created in DYRA were extracted out in each test, reduced, and utilized to transmit 10,000 VERO E6 cells that had been pre-seeded. The viral supernatants were extracted after 1 h of adsorption, new media was applied, and the cell was cultured for 24 h at 37 °C per 5% CO_2_. The IA was carried out upon cellular membranes, as previously reported. The ½ inhibition dose (IC50) was estimated using GraphPad Prism software program (version 6.01) and non-linear linear regressions of the dosage curves. Remdesivir has employed a standard drug for SARS-CoV-2 for each test. Carrying the virus and non-infected cells with medicines have been applied to compute virus assembly rates of 100% and 0%, respectively. The proportion of CC50 and IC50 was used to estimate the Sensitivity Index (SI). The upper the SI result, the greater the safety and efficacy which would be noticed while in vivo behavior [127].

L. R. Bernstein and L. Zhang (2020) provided a culture of African green monkey kidney Vero E6 cells, the American Type Culture Collection (ATCC, CRL-1586), which were verified using a microscopic morphological study and development edge detection. The mycoplasma-free culture was kept in minimum Eagle’s Medium containing (MEM) enriched to 10% foetal bovine serum (FBS) at 37 °C in a temperature-controlled container containing 5% CO_2_. A single isolate of SARS-CoV-2 (GISAID EPI ISL 402124 h; CoV-19/Wuhan/WIV04/2019) was cultured in Vero E6 cells and virus intensity was measured using an immunofluorescence test using a 50% tissue culture infective dosage (TCID50). All of the infection studies were carried out in a laboratory with a biosafety level of 3 (BSL-3). Well before Vero E6 cells (5 × 10^4^ cells per well) were pre-treated with various concentrations of GaM for 1 h before being infected with the virus at a multiple of infection (MOI) of 0.01. The cells were then cultivated with fresh GaM-containing media after the resulting virus-drug mixture was removed. A volume of 80 mL cell supernatant was obtained from each well 24 h after infection for minimal state opposite polymerase series reaction detection of antibodies RNA (qRT-PCR). All of the tests are carried out in three. Prism software was used to conduct the statistical analysis. GaM inhibited viral replication dose-dependently, with an EC50 (concentration inhibiting viral replication by 50%) of around 14 mM (CI 95% 8.9–22.8 mM) and r214 0.88 for Goodness of Fit and Slope of 0.6 (CI 95% 0.42–0.77) (Figure S5). At doses up to 200 mM, the gallium maltolate was not hazardous to the cells (the function of cells at 200 mM was 109(3)% compared with the control cells) (Figure S6). [128] The EC50 data was estimated by an inhibitor vs. balanced response, movable slope equation:

**Y = 100/(1 + 10exp((LogEC50 *−* X) *×* HillSlope)).**

The repurposing of therapeutic applications metal-based treatments is one example of a straightforward technique. The optimal candidate should be able to combine good antiviral effectiveness with low toxicity. For that purpose, we recommend a quick assessment of auranofin (Ridaura), abbreviated as AF. AF is a medication that was licensed by the FDA in 1985 to treat rheumatoid arthritis. It works by modulating the human immune system. AF has reduced toxicities and is fit for human use. The therapeutic efficacy of AF, which is almost certainly multi-target, is still unknown. However, there is general agreement that the primary target should be thioredoxin reductase 1, resulting in disruption of the basic dehydrogenases pathways, regulation of intracellular redox state, and reactive oxygen species (ROS) induction. Even though ribosome would be an important target, it is nonetheless crucial [129,130,131]. AF has sparked a lot of interest in recent years due to its versatility and ability to be reprocessed for a variety of medicinal applications, including anti-bacterial, anti-cancer, and anti-parasitic agents [129]. The significant antiretroviral activity was also observed, prompting AF to enter medical testing as an endocrine therapy drug. Its value was noticing which AF was the initiate to be more efficient than hydroxychloroquine and chloroquine in relating to many processes involved in virus-related generation, incubation, and reactivation, as well as in reducing the infected reservoir, in case of HIV infection [132]. AF, like tocilizumab, has been shown to interfere with interleukin [133] signaling by blocking JAK1 and STAT3 phosphorylation, inhibiting a small number of proteases, or to interact preferentially to free cysteine in proteins, such as metabolic enzymes [133]. We endorse the off-label fast estimation of AF in COVID-19 individuals based on these grounds. Surprisingly, an article about AF and COVID-19 was found in the public field through the analysis process of this text [134].

Scientists T. Marzo and L. Messori (2020) claimed that at a modest µm dose, AF effectively suppresses SARS-CoV-2 multiplication in living organisms, resulting in a 95% decrease in virus RNA. Furthermore, AF was instituted to significantly inhibit the SARS-COV-2-induced cytokines in living cells, which is consistent with prior findings. These clarifications back up our hypothesis, indicating that AF, with its favorable and multitarget mechanism, could be a beneficial medication for limiting the SARS-CoV-2 virus and treating related pneumonia. We suggest that further in vitro analysis of a larger screen of typical metal-based treatment comprising a multiplicity of metal centers, such as Ru and Bi, should be explored and pursued in addition to the definite recommendation of auranofin and related Au complexes. We might reasonably expect that in a few occurrences, such uncommon and distinct metal centers will have significant and beneficial impacts on such novel pathogens that are impossible to predict in advance [135].

**Figure 7:**
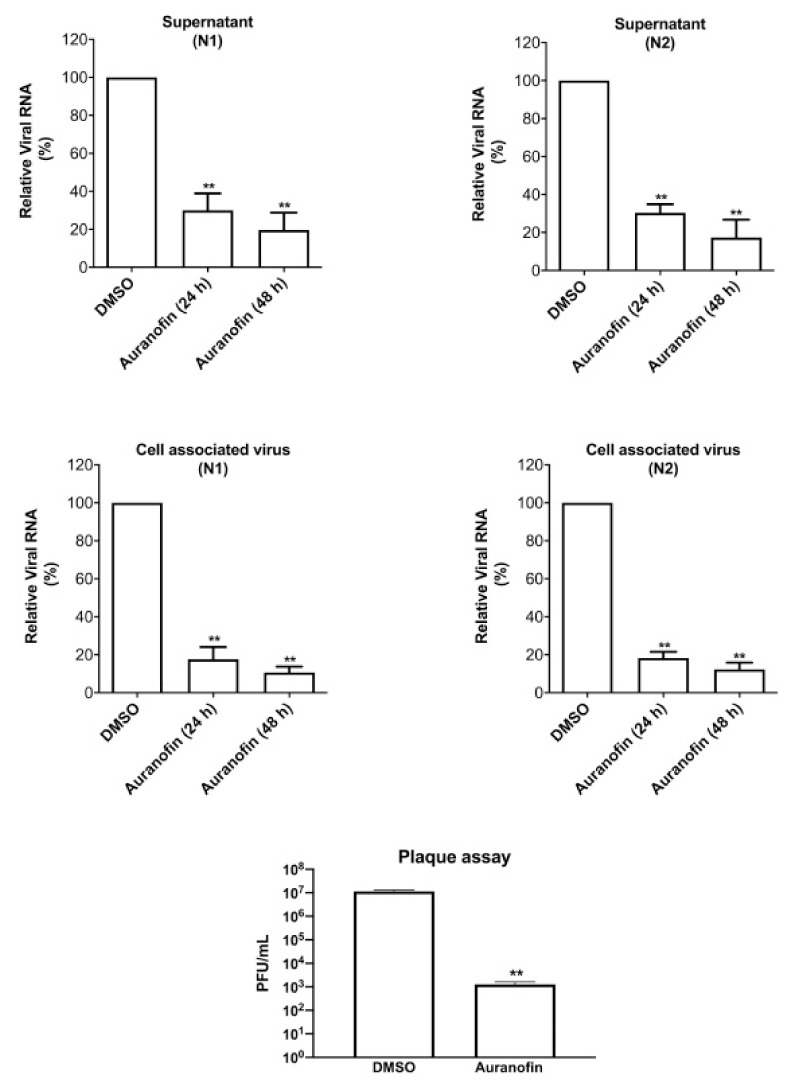
Auranofin inhibits SARS-CoV-2 proliferation in living organisms. Huh7 infection is associated with SARS-CoV-2 for 2 h at a multitude of infected (MOI) from one before ever being incubated with 4 M auranofin or 0.1 DMSO. Cell lysates and culturing filtrates were obtained 24 and 48 h after transmission, and viral RNA rates were measured using RT-PCR using primers and probe targeting the SARS-CoV-2 N1 and SARS-CoV-2 N2 regions. After measuring, standardizing, and correcting the human RNA extracted form tumor cells, the viral RNA values per ug of total cellular RNA were determined. The outcomes were compared for all specific primers, revealing a significant drop in viral RNA following 24 and 48 h. SARS-CoV-2 transmissibility titers were obtained using a cell test.48 h after invasion, in culturing filtrates The data are the mean SEM of two separate tests performed in duplicate, *t*-test ** *p* < 0.001. Reprinted/adapted with permission from Ref. [134].

**Figure 8:**
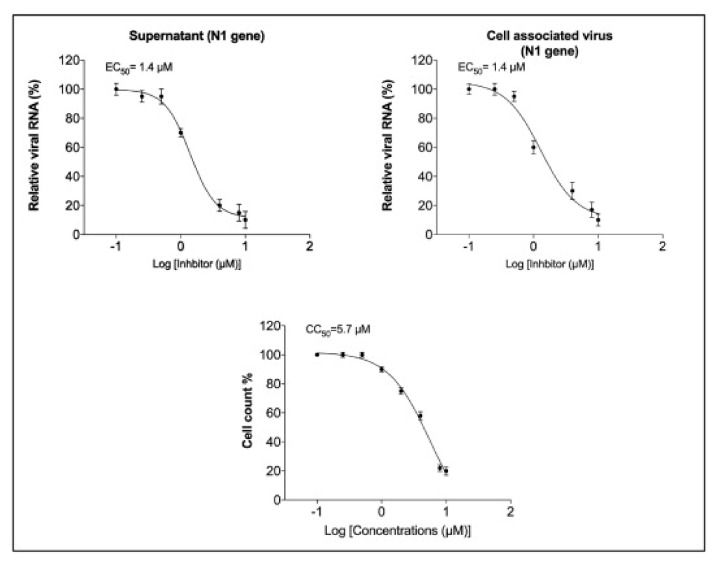
Auranofin-treated cells have a dose-dependent decrease in SARS-CoV-2 RNA: Auranofin (0.1–10 μM) was applied to SARSCOV-2 infected Huh7 cells in repeated dilutions. RT-PCR was used to quantify viral RNA in cell pellets and culture supernatants using primers and probes targeting the SARS-CoV-2 N1. The data were graphed using a non-linear regression model (GraphPad software). Auranofin reduced virus replication in infected cells with an EC50 of about 1.4 μM. The 50% cytotoxic concentration was around 5.7 μM. The data represent two separate tests that were carried out in duplicate, with permission from [134].

**Figure 9:**
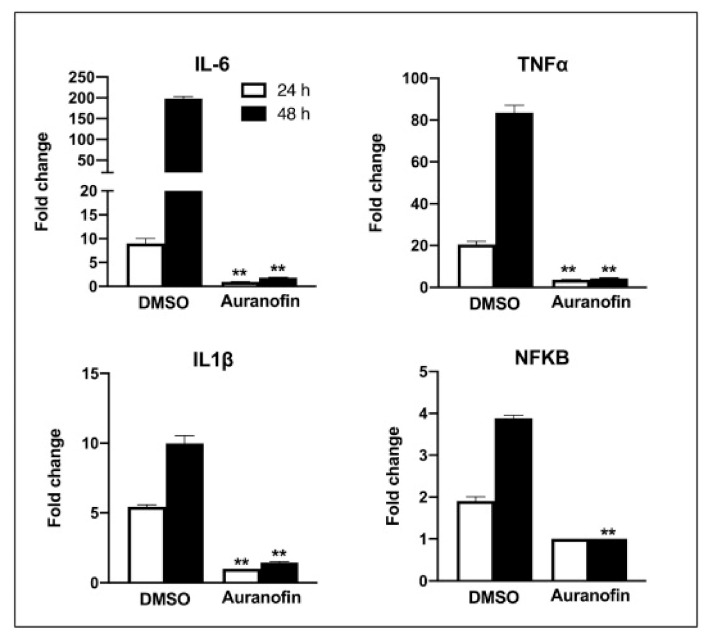
Auranofin therapy significantly inhibits the incidence of SARS-CoV-2-induced immune cells in living organisms: qRT-PCR was used to determine mRNA levels of IL-6, IL-1, TNF, and NF-kB at 24 and 48 h after transmission. Following correcting the GAPDH gene, the fold variation in tumor cells compared to baseline controls was determined. The data shows the mean ± SEM of two separate tests that were performed in parallel. ** *p* < 0.001. Reprinted/adapted with permission from Ref. [134].

Rothan et al. (2020) examined auranofin’s antiviral efficacy towards coronavirus as well its influence on disease inflammatory response in living organisms. We treated Huh7 cells towards SARS-CoV-2 (USA-WA1/2020) on 2 h at a multitude of invasion (MOI) of 1, and then added 4 M auranofin. As a control, 0.1% DMSO was employed (the solvent was utilized to synthesize drug stock). Huh7 cells were chosen for our investigation because they are very sensitive to SARS-CoV-2 replication. At 24 and 48 h following infection, Culturing supernatants and protein aggregates were obtained. RT-PCR was used to measure virus RNA copies by two different targets, another for the antiviral N1 domain and the other for the viral N_2_ region [136,137]. Auranofin treatment of cells resulted in a 70% reduction in virus RNA infiltrates relative to DMSO 24 h following the invasion, as seen in (Figure 7). When compared to DMSO, the virus RNA in the cell medium was reduced by 85% after 48 h. Similarly, as opposed to DMSO-treated cultures, the levels of intracellular viral RNA in auranofin-treated organisms reduced by 85% around 1 d and 95% around 2 d. Both primer sets had essentially identical findings. We then used a plaque test to determine viral titers in the cell culture supernatant. At 48 h after infection, auranofin treatment greatly decreased SARS-CoV-2 infectivity titers in cell culture supernatants (Figure 7). We used repeated dilutions of auranofin to treat SARS-COV-2 infected Huh7 cells in order to identify the optimum dose of auranofin that suppresses 50% of transcription and replication (EC50). After 2 d from treatment, target RNA was estimated through RT-PCR from supernatants and cell lysates. Non-linear estimation techniques were used to plot the data in graphs (GraphPad software). In auranofin-treated cells, viral RNA activity was decreased in a dose-dependent manner after 48 h. The EC50 values of auranofin therapy against Huh7 cells diseased with SARS-CoV-2 are shown in (Figure 8). At an EC50 of roughly 1.4 M, auranofin reduced viral multiplication in infected cells. It is worth noting that in comparison to formerly described results on anti-virus activity of hydroxychloroquine, chloroquine, and remdesvir towards SARS-CoV-2 in vitro, we utilized around 20 to 100 times larger pathogen dose (MOI of 1) to attack the cells in our study [138,139]. We evaluated the degree of essential proteins in auranofin and DMSO-infected cells at 1 and 2 d after treatment to see how auranofin affected the inflammatory response during SARS-CoV-2 infection [137]. In Huh7 cells, SARS-CoV-2 transmission causes a substantial upregulation of IL-6, TNF, IL-1, and NF-kB (Figure 9). In Huh7 cells, auranofin therapy effectively diminished the release of SARS-CoV-2-infected protein. In comparison with mock-infected tissues, SARS-CoV-2 complex combination in a 200-fold spike in IL-6 gene transcription 2 d after transmission. Auranofin-treated cells, on either, only had a 2-fold rise in IL-6 expression. At 2 d after transmission, TNF levels have risen 90-fold on DMSO-preserved, but not in the auranofin-treated organisms, there has been no change in the transcription of IL-1 or NF-kB [134].

## 10. Toxicity of Metals and Complex Inorganic Materials

Metal toxicity can be produced by both essential metal ions, such as iron and copper, as well as non-essential metals, such as cadmium, lead, and mercury, which are not required for life but can have toxic effects when introduced into the human environment, frequently with disastrous repercussions. So, let us start with the basics: what are essential metal ions, why are they necessary, and when are specific metal ions toxic? After that, we talk about which non-essential metal ions are hazardous to humans. General chemical concerns and the basic principles involved in metal ion toxicity are briefly discussed for each of these two classes of metal ions. Along with how the metal ion is associated within cells or tissues, as this is a vital part of developing chelation techniques to remove it. It is obvious that an excessive amount of either of these metal ions can cause a range of harmful effects. The goal of this volume in the RSC Metallobiology series is to provide a clear and up-to-date view on chelating drugs’ therapeutic potential in the control of metal excess. As much as possible, chelating agents should meet the following criteria: (i) be selective towards the metal ions that they are designed to remove (for example, Fe^3+^ can be chelated selectively from other essential transition metal ions relatively easily); (ii) causing a lot limited, if any, involvement with vital biologically important metal ions and the biochemical activities in which they have been associated [140,141,142,143]; (iii) connect possible sites in which chelation is preferred; and (iv) be emitted from the cell membrane site of complexation and successively expelled from the body without causing toxic effects (e.g., nephrotoxicity). In theory, both types of metal toxicity can be treated using proper metal ion chelators, which is the topic of this addition to the RSC Metallobiology project [144]. The toxic effects of metals are typically affected by the free metal ion’s first contact with the toxic site [145]. The toxicity of a metal-containing inorganic complex will thus be estimated by its capability to emit metal that may connect with both the targeted site. This really might be observed, though, as for some metals and inorganic complexes, the free metals must be studied, as well as some counter ions (for example, K_2_CrO_4_ in ZnCrO_4_, and PdCrO_4_), that might contribute significantly role in such unwanted consequences (e.g., local toxicity effects). Toxicity occurs when a metal poisonous molecule reaches a significant point inside the body and overcomes the body’s protection and therapeutic processes. Although each metal has distinctive properties and physicochemical properties which assign its vital trace or toxicity possible mechanisms, there have been several popular fields while evaluating the health promotion toxic effects of metal-bearing complex inorganic materials, like speciation, permeability and valence, particle size, essentiality, and interactions. These factors will influence the methods used to study the material’s behavior in a living organism (toxicokinetics), as well as outcomes, like genotoxicity and sensitization. The bioavailability of the various metal elements that complex inorganic materials can transfer into the environment affects their ecotoxicity. To summarize, bioavailability is the most important factor in predicting cytotoxic effects and should be evaluated as thoroughly as probable. It is especially true on complex inorganic molecules, somewhere simply having metal in the material does not ensure that it has the biocompatibility properties of metals. Overall toxicity is usually determined by the metal ion’s real bioavailability (i.e., its relief from the body). In the absence of biocompatibility statistics over toxicology studies in vitro, the bioaccessibility result is utilized for estimation. Once data and assays are insufficient to handle bioavailability systematically, the considerations about biocompatibility, as well as the corresponding predicted impact on outcomes, must be obviously documented in the insufficient to handle [146,147]. The toxicity of newly produced complexes was tested using the Daphnia Magna test. The crayfish were delicate to low amounts of metals corresponding to Ni, Co, Zn, Cu, and Pt. In relation to past research, the more toxic is Cu, followed by Ni, Co, Zn, and Pt. The data of the Daphnia Magna test are definite in (Table 6) [148].

## 11. Conclusions

There is no effective therapy for COVID-19 at this time, and the study of metal complexes could be useful in this regard. The majority of the studies were done in a silico approach. Docking score, active sites, the energy of frontier molecular orbitals, and definitions of the molecular structure were used to gain a theoretical understanding of synthesized ligands and their metal chelates, which were then equated to drugs primarily utilized to delicacy COVID-19, like dexamethasone, favipiravir, hydroxychloroquine, and remdesivir. Furthermore, using a molecular docking technique, the inhibitory effects of tested complexes on the basic proteolytic (6LU7) protein of SARS CoV-2 were identified, as well as the impact of linkers for anti-COVID activity. According to the results of the theoretical calculation, such molecules have a strong 6LU7 antagonistic effect on SARS-CoV-2. Our results may assist in the identification of novel ligands and their complexes, as well as their possible benefits in the detection and care of SARS-CoV-2. Such activities of transition metals should be assessed using proper in vitro and in vivo studies, according to the scientists. To avoid any unexpected effects, problems of toxicological effects should also be addressed. This review discusses some concepts about the main mechanical factors when controlling the human health and environmental concerns represented by metal complexes, for example, speciation, particle size, biocompatibility, natural background, and essentiality. It also outlines the parameters that necessitate the respect of metal specificities once evaluating the basic processes of toxicity.

## Figures and Tables

**Table 1:** Docking modeling investigations were conducted using MOE-docking score energy (DS), the sequencing of ferrocene derivatives and the references, and SARS-CoV-2 (6LU7 proteins).

Compounds	Binding Sequence	BE
L1	GLY143, CYS145	−6.00
L2	TRP218, ARG222	−5.20
L3	GLY278, ARG222, and ARG279	−4.64
L4	GLY143	−5.01
hydroxychloroquine	LEU141, SER144, HIS163, and GLU166	−5.07
remdesivir	TYR237, MET276, ASN277, and GLY278	−1.43
favipiravir	GLN74, LEU75, VAL77, VAL68, LEU67, and PHE66	−3.77
dexamethasone	THR26, ASN142, and GLU166	−6.69

**Table 2:** The energy-derived values in the ligand (HL) docking calculations were calculated using unliganded active site primary protease COVID-19 (6W41).

Receptor	6 W41
Gibbs free energy binding (kcal/mol)	−5.88
Steady inhibition (Ki) (uM)	48.92
vdW^+^ H bond + desolv energy (kCal/mol)	−7.09
Energy of electrostatic (kCal/mol)	−0.06
Total intermolecular energy (kCal/mol)	−7.15
Interaction at the surface	603.803

**Table 3:** The docking of PDBS, CPBS, (FePDBS (H2O) NO_3_), and (FeCPBS (H_2_O)NO_3_) with the active sites of the COVID-19 major protease viral protein receptor was studied (PDB ID: 6lu7).

Interaction		Distance (Å)	Receptor	E (kcal/mol)
**CPBS**				
O 23	H-donor	2.94 (1.96)	O LEU 287	−4.2
Br 26		3.35	O THR 169	−1.5
6-ring	pi-H	3.51	CE LYS 137	−0.5
		3.97	CB ASP 197	−0.7
**[FeCPBS(H_2_O)NO_3_]**				
O 37	H-donor	2.79 (1.77)	OD1 ASN 142	−15.7
Br 27		3.50	O THR 26	−0.7
O 40	H-acceptor	3.10 (2.17)	NE2 GLN 189	−1.1
6-ring	pi-H	3.96	CA ASN 142	−0.5
**PDBS**				
O 24	H-donor	2.92 (1.94)	O THR 196	−3.5
6-ring	pi-H	4.80	CB PRO 132	−0.6
		3.87	CA ASN 133	−0.5
**[FePDBS(H_2_O)NO_3_]**				
O 37	H-donor	2.80 (1.90)	O LEU 272	−13.3

**Table 4:** Docking calculations of H2L and its metal complexes with to Crystal structure of the SARS-CoV-2 (COVID-19) primary protease in complex with inhibitor UAW247 (6XBH).

Compound	Distance (Aο)	Moiety	Interaction	Receptor Site	E (kcal/mol)
**Ligand (H_2_L)**	2.88	O 16	H-donor	OE2 GLU 166	−2.5
	3.45	C2 26	H-donor	SD MET 165	−1.1
	3.36	5-ring	pi-H	NE2 GLN 189	−0.9
**(Fe(H_2_L)(H_2_O)_2_Cl)Cl_2_**	2.86	O 15	H-donor	O GLU 166	−5.0
	2.91	O 54	H-donor	OD1 ASN 142	−3.6
**(Cr(H_2_L)(H_2_O)_2_Cl)Cl_2_-2H_2_O**	3.09	O15	H-donor	O THR 24	−1.0
	3.38	O54	H-donor	SD MET 49	−28.6
	4.55	6-ring	pi-H	CG2 THR 25	−0.7
**(Cu(H_2_L)Cl)Cl** **-** **2H_2_O**	2.88	O 15	H-donor	O ASP 187	−2.9
	3.34	CL 50	H-acceptor	CG GLN 189	−0.8
	3.08	CL 50	H-acceptor	NE2 GLN 189	−3.0
**(Mn(H_2_L)(H_2_O)_2_Cl)Cl-2H_2_O**	4.03	O 22	H-donor	SD MET 165	−0.4
	3.47	CL45 49	H-donor	SD MET 49	−3.1
	3.07	O46 50	H-donor	SD MET 49	−0.9
	2.70	O 54	H-donor	OG1 THR 25	−1.4
	3.77	CL45 49	H-acceptor	NE2 GLN 189	−3.3
	3.92	5-ring	pi-H	CG2 THR 25	−0.7
	3.64	6-ring	pi-pi	5-ring HIS 41	−0.0
**(Zn(H_2_L)(H_2_O)Cl_2_)-2H_2_O**	4.24	CL45 49	H-acceptor	CA ASN 142	−0.6
	4.03	CL46 50	H-acceptor	CA ASN 142	−0.9
	3.06	CL46 50	H-acceptor	N GLY 143	−8.0
	4.27	CL46 50	H-acceptor	N CYS 145	−1.9
	3.60	CL46 50	H-acceptor	SG CYS 145	−0.9
	4.80	6-ring	pi-H	N GLU 166	−0.7
**(Ni(H_2_L)(H_2_O))Cl_2_-4H_2_O**	3.31	O 50	H-donor	SD MET 49	−4.9
**(Cd(H_2_L)(H_2_O)Cl_2_)**	3.51	O 15	H-donor	SD MET 165	−2.6
	3.92	CL45 49	H-donor	SG CYS 145	−3.8
	3.48	CL45 49	H-acceptor	CA MET 165	−2.2
	4.16	CL45 49	H-acceptor	N GLU 166	−1.1
	3.34	CL46 50	H-acceptor	N GLU 166	−6.5
	3.64	CL46 50	H-acceptor	CB GLU 166	−0.8

**Table 5:** Docking tests in (kcal/mol) and estimated binding properties for compounds 4a–4k with SARS-CoV-2 main protease were calculated.

Compound	Main protease (Mpro)	
	Docking Score (kcal/mol)	Binding Features (Hydrogen Bond Length in Å)
**4a**	−9.1	HIS163 (1.97), SER144, GLU166 (1.89 Å), (2.31 Å), and LEU141 (1.97 Å)
**4b**	−9.0	SER144 (2.24 Å), GLU166 (2.15 Å), and LEU141 (1.99 Å)
**4c**	−7.7	GLU166 (2.13 Å), ARG188 (1.82 Å), and MET165 (2.52 Å)
**4d**	−9.7	SER144 (2.22 Å), LEU141, GLU166 (2.07 Å), (2.02 Å), and HIS163 (1.83 Å)
**4e**	−7.5	GLU166 (2.13 Å)
**4f**	−7.9	GLN189 (2.23 Å), and GLY143 (1.75 Å)
**4g**	−8.2	GLU166 (2.32 Å), and GLN192 (1.92 Å)
**4h**	−8.5	GLU166 (1.86 Å), and GLN192 (1.99 Å)
**4i**	−8.6	GLU166 (1.86 Å), GLN192 (2.24 Å), SER144 (2.21 Å), and CYS145 (2.37 Å)
**4j**	−8.7	GLU166 (2.21 Å), and GLN192 (2.19 Å)
**4k**	−8.8	GLU166 (2.02 Å), HIS163 (1.93 Å), and GLN192 (2.22 Å)

**Table 6:** Toxicity of HL, metal salts, and newly created complexes on Daphnia magna.

Compound	Incubation Period					
	1 d	2 d	d1	2 d	1 d	2 d
	LC50 (µM)		Goodness of Fit (r2)		CI 95% of LC50 (µM)	
**HL**	53.09	52.60	0.9918	0.9849	ND*	ND*
**(Cu(L)(CH_3_COO)).2H_2_O**	69.59	13.09	0.9021	0.9389	44.11–109.8	8.89–19.26
**(Cu(L)(NO_3_)).H_2_O**	316.90	5.61	0.7842	0.8742	2.19–2.81	0.51–0.98
**(Cu(L)(ClO4)).H_2_O**	ND*	ND*	ND*	ND*	ND**	ND*
**(Cu(L)(Cl)).2H_2_O**	393.8	69.53	0.9966	0.8159	374.4–414.2	33.96–142.4
**(Co(L)_2_)**	278.2	2.38	0.8655	0.9145	166.4–465.4	158.4–265.8
**(Zn(L)_2_)**	33.91	0.84	0.9452	0.7553	23.27–49.4	0.33–2.12
**(Ni(L)(Cl))**	350.8	209.7	0.9935	0.9315	320.6–383.8	149.6–294.1
**(Pt(L)(Cl))**	468.60	78.19	0.9490	0.9546	400.6–548.3	57.44–106.5
**Cu(CH_3_COO)_2_**	9.86	0.44	0.9980	ND*	ND**	ND**
**CuCl_2_**	4.23	0.97	0.9890	0.9302	3.709–4.815	ND**
**Cu(NO_3_)_2_**	7.43	1.39	0.9586	0.9446	6.04–915	1.08–1.78
**Cu(ClO_4_)_2_**	0.39	0.31	0.9777	0.9794	2.34–3.76	0.99–1.22
**CoCl_2_**	205.20	31.37	0.9717	0.876	158.4–265.8	17.06–57.7
**ZnCl_2_**	15.39	0.89	0.9190	0.9691	9.42–25.15	0.79–1.02
**NiCl_2_**	100	16.25	0.9827	0.9624	98.31–101.7	11.86–22.27
**K_2_PtCl_4_**	27.70	5.63	0.9918	0.9364	21.86–35.11	3.953–8.012

ND*—not detected since the largest L% is 10% following 1 d and 35% following 2 d, respectively; ND**—not detected because of CI 95% is really wide.

## Data Availability

The raw/processed data generated in this work are available upon request from the corresponding author.

## Supplementary Materials

The following supporting information can be downloaded at: https://www.mdpi.com/article/10.3390/ijms23126418/s1.
