# Supplementary material for: Development of Metal Complexes for Treatment of Coronaviruses
Source: Int J Mol Sci. 2022 Jun 8;23(12):6418. doi: 10.3390/ijms23126418 (PMC9223400; doi:10.3390/ijms23126418)
Supplement: Supplementary file 1 [file ijms-23-06418-s001.zip › ijms-1737672-supplementary.pdf]

International Journal of
Molecular Sciences

Article

Development of Metal Complexes for Treatment of Corona Viruses

Hany M. Abd El-Lateef [1,2,*], Tarek El-Dabea[2], Mai M. Khalaf [1,2], and Ahmed M. Abu-Dief [2,3,*]

[1] Department of Chemistry, College of Science, King Faisal University, P.O. Box 400 Al-Ahsa 31982, Saudi Arabia; hmahmed@kfu.edu.sa(H.M.A.); mmkali@kfu.edu.sa (M.M.K.)

[2] Chemistry Department, Faculty of Science, Sohag University, Sohag-82534, Egypt; (T.A.) tarekeldabea@gmail.com

[3]Chemistry Department, College of Science, Taibah University, Madinah, P.O. Box 344, Saudi Arabia; amamohammed@taibahu.edu.sa (A. M. Abu-Dief)

* Correspondence: Corresponding authors: hmahmed@kfu.edu.sa (H. M. Abd El-Lateef); amamohammed@taibahu.edu.sa; ahmed_benzoic@yahoo.com (A. M. Abu-Dief)

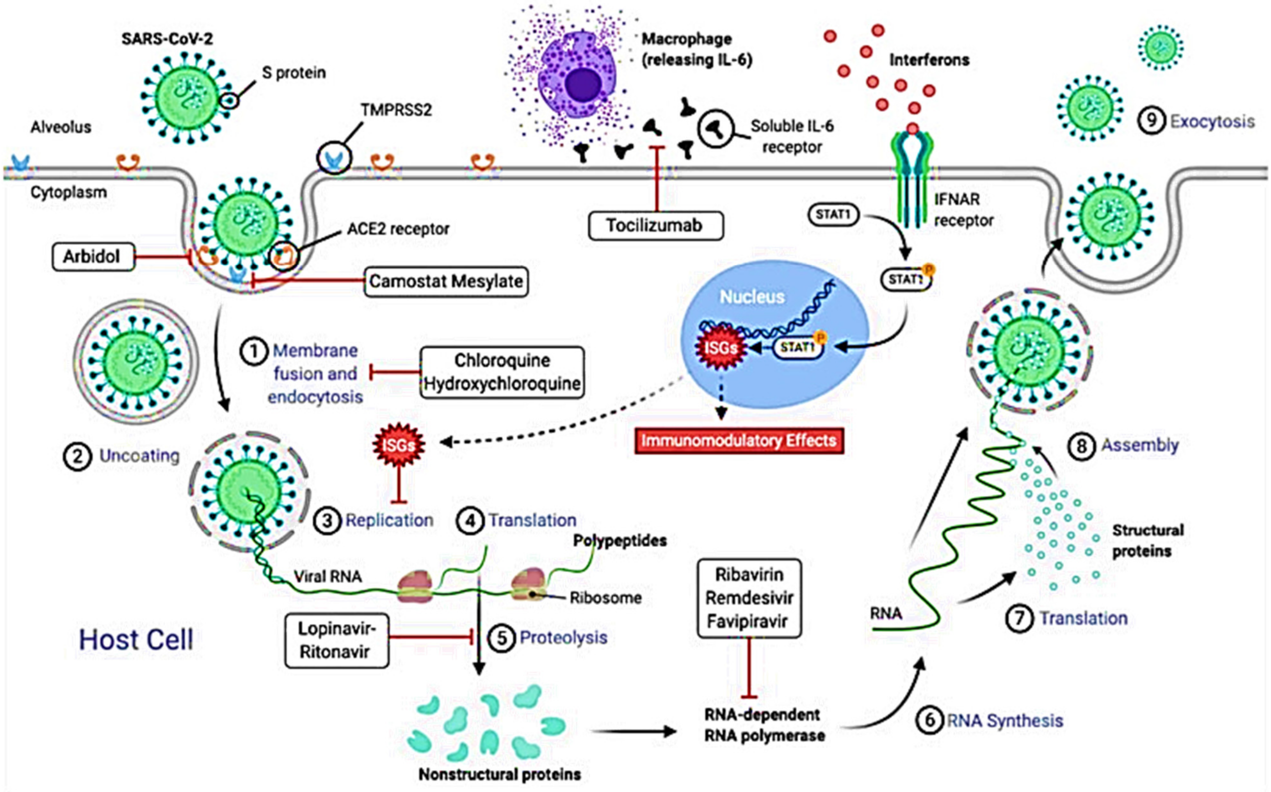

Figure S1. Mechanisms of SARS- Coronavirus 2 transmittion cycle & various therapy drugs for dealing of COVID-19. TMPRSS2: 2 Trans membrane serine protease; S protein: Spike protein ACE2: Angiotensin converting enzyme 2; IL 6: RNA: Ribonucleic acid; Interleukin 6; ISGs: Interferon-stimulated genes; STAT1: Signal transducer; IFNAR: Interferon-α/β receptor; P: Phosphorus and activator of transcription 1.

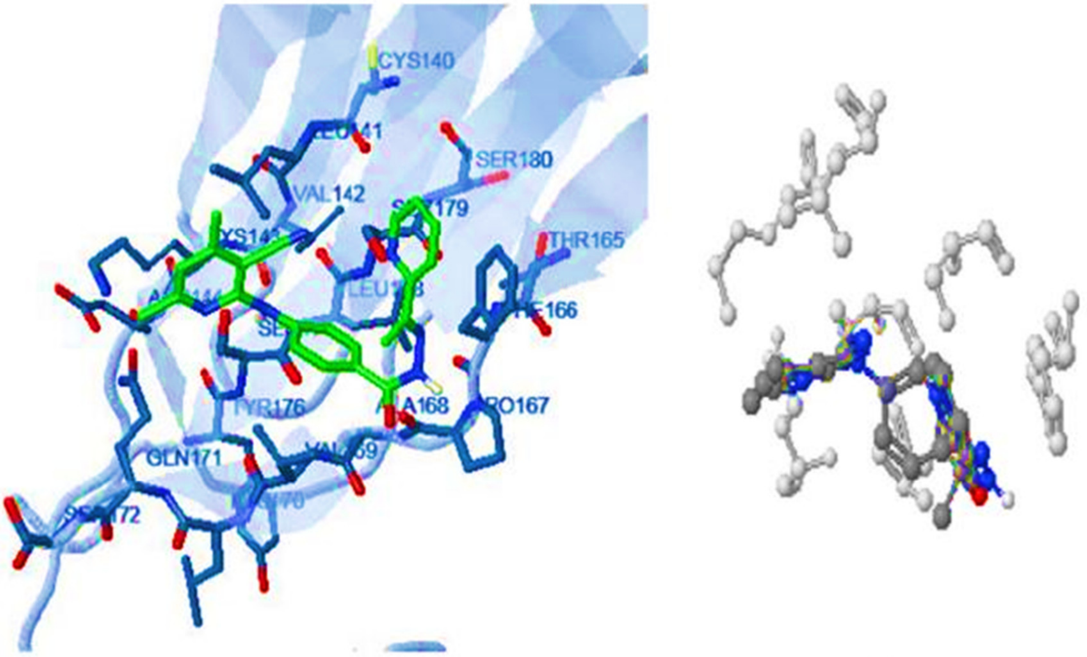

(a) (b)

Figure S2. Ligand (HL) containing 6 W41 SARS-CoV-2 major protease association with non-liganded COVID-19 protein surface, shown in green in (a) and blue in (b) [87].

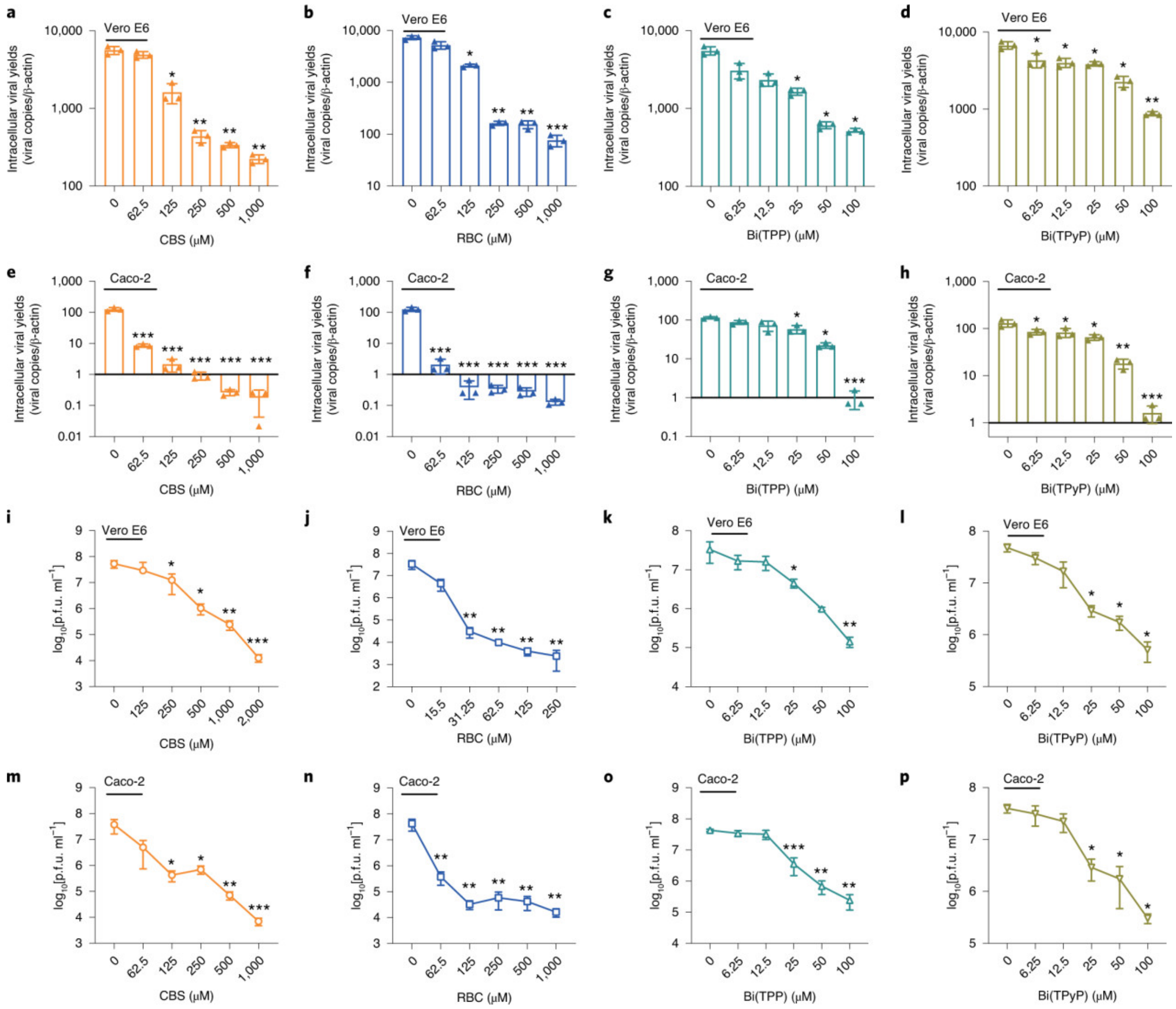

Figure S3. The antiviral activity of the evaluated drugs in vitro Caco-2 and Vero E6 cells were infected with SARS-CoV-2 (MOI = 0.1) and maintained with varied doses of RBC and tested chemicals. a–h, Intracellular virus loads were detected 48 hours after infection and were regulated by human -actin in Caco-2 cells (e–h) and Vero E6 cells (a–d). i–p, Virus replicas in cell culture media were identified at 48 hours post infection (h.p.i) using a qualitative polymerase chain reaction with opposite transcription (qRT–PCR) in Caco-2 cells (m–p) and Vero E6 cells (i–l). On metal contented, drug concentration processes were developed. All effects are presented as mean s.d. An unpaired two-tailed Student's t-test (n = 3) was used to calculate statistical significance; *P< 0.05, **P< 0.01 and ***P< 0.001 once connected with vehicle control (0 M). All tests were completed in duplicate and repeated twice for verification [124].

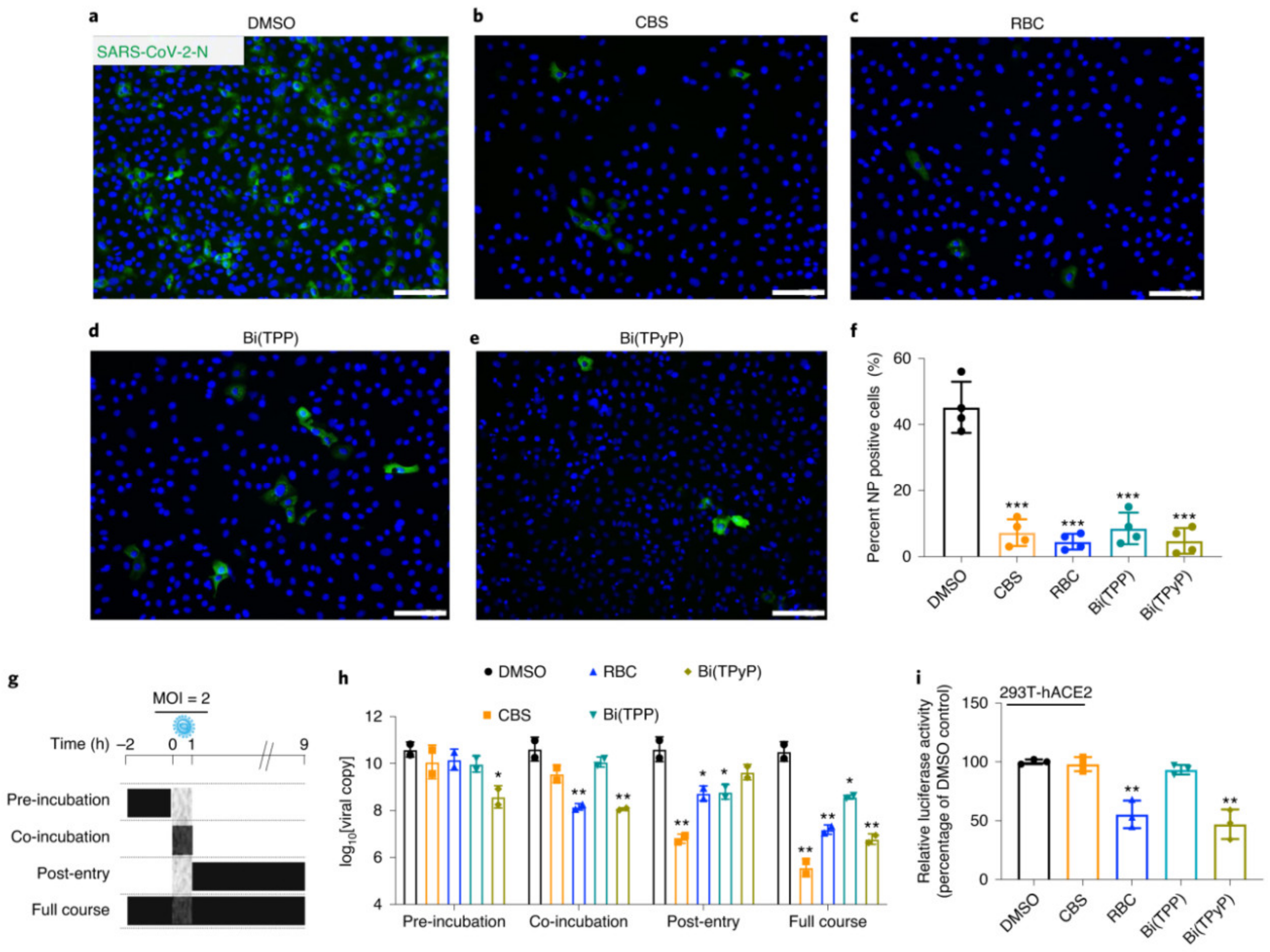

Figure S4. Antiviral compound action phases, a–e Immunofluorescence labelling photographs revealing multiple medicinal compounds' anti-SARS-CoV-2 effects. Vero E6 cells infected with SARS-CoV-2 (MOI = 0.1) were fixed and stained after 24 hours of treatment with DMSO control (a), CBS (b), RBC (c), Bi(TPP) (d), and Bi(TPyP) (e). In blue and green, antigens of the SARS-CoV-2 nucleocapsid protein (SARS-CoV-2-N) and cell nuclei (DAPI) were detected. Scale bars at 100 m. f. In two independent experiments, antigen-positive cells were counted in 800 × 800 pixel fields (n = 4). (ANOVA, one-way analysis of variance) SARS-nucleocaspid CoV-2 protein is denoted by the abbreviation NP. g,h, A time-of-drug-addition experiment was used to determine which phases of the viral replication cycle were targeted by each of the four therapeutic agents.The experimental design is presented in g, which reflects the length of incubation of the cell-compound. After 0–1 h (MOI = 2) of viral absorption, fresh medium supplemented with the tested medication or DMSO was replaced. The infection yields in all groups' cultures were measured by qRT–PCR at 9 (h.p.i) in h bar charts. Using one-way ANOVA, the treatment groups were compared to the negative control group (0 M, 0.1 percent DMSO). In the presence of DMSO or pharmacological agents, I pseudo-SARS-CoV-2-Luc was infected into HEK293-hACE2 stable cells (n = 3). Luciferase activity was measured and normalised 48 hours after injection as a proportion of the DMSO control. A one-way ANOVA was used. Medication concentration was determined using metal content. All effects are presented as the mean (s. d.) of the number of biological repeats indicated. An unpaired two-tailed Student's t-test, *P <0.05, **P< 0.01 and ***P< 0.001 were used to determine statistical significance. For verification, all tests were pretended twice [124].

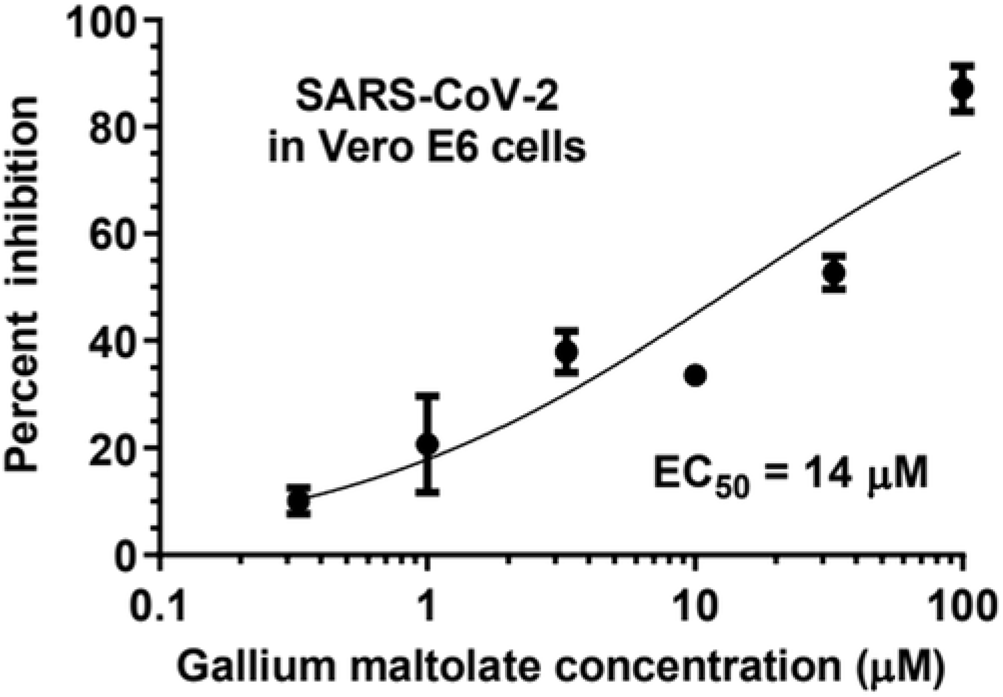

Figure S5. Gallium maltolate suppresses SARS-CoV-2 replication in Vero E6 cells at various concentrations, [128]

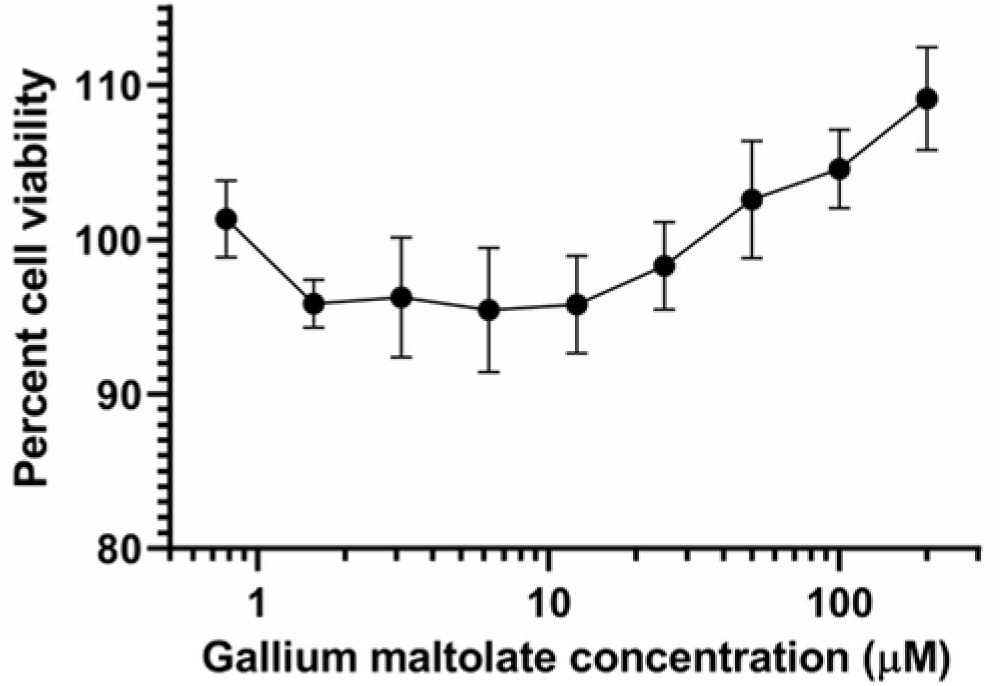

Figure S6. Vero E6 cell viability at different gallium maltolate concentrations [128].

References

[87] El-Gammal, O. A., El-Bindary, A. A., Mohamed, F. S., Rezk, G. N., & El-Bindary, M. A., J. Mol. Liq, 2022,346, 117850.

[124] Yuan, S., Wang, R., Chan, J. F. W., Zhang, A. J., Cheng, T., Chik, K. K. H., ... & Sun, H. Nat microbiol, 2020,5(11), 1439-1448.2020

[128] Bernstein, L. R., & Zhang, L., Antivir. Chem. Chemother, 2020,28, 2040206620983780. https://doi.org/10.1177%2F2040206620983780
